# ClC-3/SGK1 regulatory axis enhances the olaparib-induced antitumor effect in human stomach adenocarcinoma

**DOI:** 10.1038/s41419-020-03107-3

**Published:** 2020-10-22

**Authors:** Zhuoyu Gu, Liping Wang, Xiaohan Yao, Qian Long, Kaping Lee, Jieyao Li, Dongli Yue, Shuangning Yang, Yanfen Liu, Na Li, Yixin Li

**Affiliations:** 1 Medical Research Center, The First Affiliated Hospital of Zhengzhou University, Zhengzhou University, Zhengzhou, China; 2 Department of Clinical Oncology, The First Affiliated Hospital of Zhengzhou University, Zhengzhou University, Zhengzhou, China; 3 Sun Yat-sen University Cancer Center, State Key Laboratory of Oncology in South China, Collaborative Innovation Center of Cancer Medicine, Guangzhou, China; 4 Department of Cardiovascular Medicine, Qingdao No. 9 People’s Hospital, Shandong, China

**Keywords:** Drug development, Gastric cancer, Prognostic markers, Translational research

## Abstract

Currently, only a few available targeted drugs are considered to be effective in stomach adenocarcinoma (STAD) treatment. The PARP inhibitor olaparib is a molecularly targeted drug that continues to be investigated in BRCA-mutated tumors. However, in tumors without BRCA gene mutations, particularly in STAD, the effect and molecular mechanism of olaparib are unclear, which largely restricts the use of olaparib in STAD treatment. In this study, the in vitro results showed that olaparib specifically inhibited cell growth and migration, exerting antitumor effect in STAD cell lines. In addition, a ClC-3/SGK1 regulatory axis was identified and validated in STAD cells. We then found that the down-regulation of ClC-3/SGK1 axis attenuated olaparib-induced cell growth and migration inhibition. On the contrary, the up-regulation of ClC-3/SGK1 axis enhanced olaparib-induced cell growth and migration inhibition, and the enhancement effect could be attenuated by SGK1 knockdown. Consistently, the whole-cell recorded chloride current activated by olaparib presented the same variation trend. Next, the clinical data showed that ClC-3 and SGK1 were highly expressed in human STAD tissues and positively correlated (*r* = 0.276, *P* = 0.009). Furthermore, high protein expression of both ClC-3 (*P* = 0.030) and SGK1 (*P* = 0.006) was associated with poor survival rate in STAD patients, and positive correlations between ClC-3/SGK1 and their downstream molecules in STAD tissues were demonstrated via the GEPIA datasets. Finally, our results suggested that olaparib inhibited the PI3K/AKT pathway in STAD cells, and up-regulation of ClC-3/SGK1 axis enhanced olaparib-induced PI3K/AKT pathway inhibition. The animal experiments indicated that olaparib also exerted antitumor effect in vivo. Altogether, our findings illustrate that olaparib exerts antitumor effect in human STAD, and ClC-3/SGK1 regulatory axis enhances the olaparib-induced antitumor effect. Up-regulation of the ClC-3/SGK1 axis may provide promising therapeutic potential for the clinical application of olaparib in STAD treatment.

## Introduction

Gastric cancer (GC) is one of the most common malignant tumors worldwide. Nowadays, the incidence and mortality of GC are 5.7% and 8.2%, ranking the fifth and third in all human cancers^1^. China accounts for 40% of new global GC cases, with 10.3% of the incidence and 12.5% of the mortality, which continues to be a global public health problem^2^. Stomach adenocarcinoma (STAD) is the most common histopathological type of GC, characterized by rapid growth and strong invasiveness. To date, the prognosis of patients with advanced STAD remains poor. Consistently, only a few targeted drugs, such as trastuzumab and ramucirumab, have been effective in clinical trials^3^, so the identification of further available targeted drugs for STAD is imperative.

Olaparib, an inhibitor of poly (ADP-ribose) polymerase (PARP) enzymes, is the most studied PARP inhibitor and continues to be investigated. In recent studies, olaparib has been effectively used as an anticancer agent and is mainly evaluated in BRCA-associated tumors^4^. Clinically, olaparib is approved in advanced BRCA-mutated ovarian cancer, advanced BRCA-mutated, HER2-negative breast cancer, and as maintenance therapy for platinum-sensitive ovarian cancer^5,6^. The potential antitumor mechanisms of olaparib are mostly involved in BRCA-mutated tumors, including transcriptional regulation, cell apoptosis promotion, and DNA repair abnormality^7–9^. However, the role of olaparib and its specific molecular mechanism in tumors without BRCA gene mutations, especially in digestive system tumors, has not been fully elucidated. Until now, the action mechanism and clinical application value of olaparib in STAD remain unclear, which is of importance to the current targeted therapy of STAD patients.

Ion channels are a class of membrane proteins that are aberrantly expressed in multiple tumor types. In addition to regulating various aspects of cancer cell behavior, ion channels are also expected to be the promising cancer biomarkers. For example, the Ca^2+^-activated chloride channel TMEM16A contributes to cancer cell proliferation and migration, suggesting that TMEM16A can be used as a prognostic marker^10^. Furthermore, the KCa3.1 potassium channel exerts pivotal oncogenic functions in tumorigenesis, malignant progression, angiogenesis, and therapy resistance^11^. However, few studies have focused on exploiting ion channels for clinical purposes in STAD treatment. Chloride channel-3 (ClC-3) is a member of the chloride channel superfamily. It is a multifunctional protein with crucial roles in the regulation of ion homeostasis, vesicle acidification, and membrane excitability^12,13^. Recent studies have shown that ClC-3 chloride channel activation is closely related to multiple processes of carcinogenesis, including apoptosis, autophagy, cell cycle, and tumor multidrug resistance^14,15^. Especially in nasopharyngeal carcinoma, opening of the ClC-3 chloride channel in the cell membrane promoted the cellular uptake of anticancer drugs, including paclitaxel and doxorubicin^16,17^. We hypothesize that the ClC-3 chloride channel may be involved in the regulation of drug transport and affect the sensitivity of cells to olaparib. However, to date the role of ClC-3 chloride channel in the drug therapy of digestive tract cancers, including STAD, has rarely been reported. Serum/glucocorticoid-regulated kinase 1 (SGK1) is a serine/threonine protein kinase that is widely expressed in many cell types. This kinase activation is involved in the regulation of processes such as cell survival, material transport, gene transcription, and neuronal excitability^18,19^. But few studies have focused on the relationship between ClC-3 chloride channel and SGK1 in tumors. Particularly in STAD, whether the ClC-3 and SGK1 are potential therapeutic molecular targets for olaparib is unknown.

In this study, SGK1 was identified and validated as the downstream target of ClC-3, and the role of ClC-3/SGK1 regulatory axis in olaparib-induced antitumor effect and the potential molecular mechanism were deeply investigated. The aim of this study was to explore the effect and molecular mechanism of olaparib in STAD cells, thus providing a theoretical basis for the study of molecular-targeted drug therapy in STAD patients and further expanding the clinical application space of olaparib in STAD treatment.

## Materials and methods

### Cell culture and construction of stable cell lines

Human STAD cell lines (SGC-7901 and BGC-823 AGS) were obtained and authenticated from the Cell Bank of the Chinese Academy of Sciences (Shanghai, China) and cultured in RPMI-1640 medium supplemented with 10% fetal bovine serum, 100 units/ml penicillin, and 100 μg/ml streptomycin. All cells were maintained in an incubator with a humidified atmosphere of 95% air and 5% CO_2_ at 37 °C. The PARP inhibitor olaparib (AZD2281, Ku0059436) was purchased from Selleck. The lentiviruses for ClC-3 knockdown (KD) (shClC-3-1, shClC-3-2), flag-tagged ClC-3 overexpression (OV) (ClC-3) and SGK1 KD (shSGK1) were purchased from Shanghai GenePharma Co. (Shanghai, China). The 5′–3′ sequences of shClC-3-1 and shClC-3-2 were designed as CCTACCTCTTTCCAAAGTATA and CCGATTAAATGGATACCCTTT, respectively. The 5′–3′ sequence of shSGK1 was designed as CTGGAAGCTTAGCAATCTTAT. SGC-7901 and BGC-823 cells were transfected using above lentiviruses according to the manufacturer’s instructions. After selection with 1 µg/ml puromycin for 4 weeks, stable cell lines were established. Negative control shRNA cells (sh-NC) and empty vector-transfected cells (vector) were established as controls.

### RNA extraction and quantitative RT-PCR (qRT-PCR)

Total RNA was extracted using a RaPure Total RNA Micro Kit (Magen, Guangzhou, China). Endogenous cDNAs were generated using the ReverTra Ace^®^ qPCR RT Master Mix kit (ToYoBo, Shanghai, China). The primers for qRT-PCR were purchased from GeneCopoeia, Inc. (Rockville, MD, USA) and are shown in Table S1. qRT-PCR was performed with SYBR^®^ Green Real-time PCR Master Mix (ToYoBo, Shanghai, China) in a Bio-Rad CFX96 PCR system. Relative mRNA expression was normalized to GADPH expression. The fold change in relative expression of mRNAs was calculated using the 2^−ΔΔCt^ methods.

### Whole-transcriptome RNA sequencing

Firstly, total RNA was isolated from cell samples and ribosomal RNA was depleted. Next, first strand and directional second strand synthesis were performed. Then the A tailing and adapter ligation were performed with the purified cDNA. Finally, the purified, adapter-ligated DNA was amplified. The library quality and concentration was evaluated and sequenced by RIBOBIO (Guangzhou, China). Each library was diluted to a final concentration of 10 nM and pooled equimolar prior to clustering. The resultant libraries were then sequenced by Illumina MiSeq (Illumina, San Diego, CA). The threshold value of differentially expressed mRNA was set by log_2_ (fold change) > 1 and *q*-value < 0.001. Trimmed mean of *M*-values (TMM) was used to normalize the gene expression. Differentially expressed genes were identified using the edgeR program.

### Antibodies and western blotting (WB)

Proteins were extracted using RIPA lysis buffer supplemented with 1% proteinase inhibitor and quantified by a BCA kit (Thermo, USA). Equal amounts of protein lysates were separated by SDS–PAGE and transferred onto polyvinylidene difluoride membranes. The membranes were blocked with 5% nonfat milk for 2 h at room temperature and then incubated with primary antibody at 4 °C overnight. The protein bands were then incubated with HRP-conjugated goat anti-mouse secondary antibody and detected by enhanced chemiluminescence. The density of the protein bands was quantified by ImageJ software (National Institutes of Health, Bethesda, MD) and normalized to GAPDH. Relative protein levels were calculated as the density ratios of the interest protein to GAPDH. All antibodies used for WB were purchased from Cell Signaling Technology (Danvers, MA, USA). All experiments were performed in triplicate.

### MTS assay and clone formation assay

Cell proliferation was determined by the MTS assay (Promega, Madison, WI). Cells were seeded at a density of 5000 cells per well in a 96-well microplate. After stimulation of olaparib for 24 and 48 h, the cells were incubated with 10 μl MTS for 40 min, and the optical density (OD) was detected at 490 nm with an enzyme immunoassay analyzer. In the clone formation assay, cells were seeded at a density of 500 cells per well in six-well plates and incubated. After stimulation of olaparib for 1 week, the cells were fixed with 4% paraformaldehyde and stained with crystal violet. The images of the clones were captured, and the numbers of the clones were counted by Image-Pro Plus 6.0 software (Media Cybernetics, CA, USA).

### Cell cycle analysis

Flow cytometry was used to analyze the cell cycle distribution. After stimulation of olaparib for 36 h, STAD cells were digested with 0.25% trypsin and collected by centrifugation at 200×*g* for 5 min at 4 °C. Then the cells were washed with cold phosphate-buffered saline (PBS) and incubated in ice-cold 70% ethanol at 4 °C overnight. Next, cells were incubated with propidium iodide (BD, USA) for 30 min and analyzed for cell cycle distribution using a flow cytometer (EPTCSXL-31240, Coulter, USA). The data are presented as the percentage of cell phase distribution including G0/G1, S and G2/M phases.

### Migration and invasion assays

Wound healing assays and transwell invasion assays were performed to determine the migration and mobility of STAD cells. Briefly, cells were cultured in six-well plates until confluence and scratched with a 10-μl pipette tip. Cell migration images were captured at 0 and 36 h. The widths of the gap at 0 h (*w*1) and 36 h (*w*2) were measured, and the relative migration rate was calculated as (*w*1−*w*2)/*w*1*100%. Cell invasion assays were performed in transwell chambers (8-μm pore size, Corning, NY, USA) precoated with matrigel (BD, NJ, USA). After stimulation of olaparib for 24 h, 2 × 10^5^ STAD cells incubated with serum-free RPMI-1640 were added to the upper chamber and RPMI-1640 with 10% FBS was added to the lower chamber. The chambers were then incubated for 24 h before examination. The cells on the upper surface were removed, while the cells on the lower surface were fixed with methanol for 20 min and stained with 1% crystal violet. Finally, cells were counted under a microscope.

### Whole-cell patch-clamp recording

The solutions in patch-clamp pipettes were described previously^20^. Briefly, the coverslip with SGC-7901 cells was placed in a microchamber mounted on an inverted microscope. Standard whole-cell patch-clamp recordings were carried out using 5–10 MΩ pipettes and a List EPC-7 patch clamp amplifier (List Electronic, Darmstadt, Germany). Cells were held at a holding potential of 0 mV (close to the chloride equilibrium potential) and then cycled by voltage steps of 0, ±40, and ±80 mV for a 200 ms duration, with a 4 s time interval between pulses. Then cells were continuously cycled through the voltage protocol in the experiments. The pulse generation and whole cell chloride currents were recorded by a computer through a laboratory interface (CED 1401, Cambridge, UK) with a sampling rate of 3 kHz and were analyzed by the EPC software package (CED, Cambridge, UK). When the basal chloride current levels were stable with no fluctuations, 20 μM olaparib was added into the extracellular isotonic (300 mosmol/l) conditions, forming an extracellular stimulation to the cells. We then assessed the effect of olaparib on chloride current formation in the membrane. The typical time courses and current traces of the chloride currents were drawn using Sigmaplot software (Systat software, CA, USA). All current measurements were collected at 10 ms following the onset of each voltage step. The current density of the whole-cell current was calculated by the following equation: whole-cell current/cell capacitance (pA/pF).

### In vivo tumor model and tissue processing

Female BALB/c nude mice (4 weeks old) were purchased from Vital River Laboratory Animal Technology Co., Ltd. (Beijing, China) and quarantined for one week before use in tumor formation experiments. All animal experimental procedures were approved by the Animal Care and Use Committee of Zhengzhou University, and every effort was made to reduce the suffering of animals. Approximately 2 × 10^6^ cells in 100 μl of PBS were subcutaneously injected to establish STAD tumor xenografts, and then the nude mice were treated with olaparib (25 mg/kg/d, intraperitoneal injection) and DMSO for 4 weeks. The volumes of the tumors were measured and recorded every 5 days according to the equation volume = (width^2^ × length)/2. At the end, the mice were sacrificed, and the tumors were excised, weighed, fixed and embedded in paraffin for immunohistochemistry (IHC) staining.

Human STAD tissue microarrays of 90 cases and matched noncancerous tissues were purchased from Superchip (HStm-Ade180Sur-04, Shanghai, China). The expression of ClC-3 and SGK1 was detected by IHC in tissue microarray slides. The slides were incubated with anti-ClC-3 and anti-SGK1 primary antibodies at a dilution of 1:100 overnight. After washing, the sections were incubated with horseradish peroxidase-conjugated anti-goat antibodies and stained with 3,5-diaminobenzidine (DAB). Scoring of tissue slides was performed independently by two investigators. For the IHC score, the percentage (0–100%) of stained tumor cells was multiplied by the intensity (0, 1, 2, or 3) to achieve a score between 0 and 300, of which 100 or higher was considered to indicate high ClC-3/SGK1 expression. The study was approved by the institutional human ethics committee of the relevant institutions.

### Statistical analysis

Statistical analyses were performed using the SPSS statistical software package (version 17.0). All data was presented as the mean ± SD. The significance of differences was assessed using two-tailed Student’s *t* test or variance analysis. Correlations between ClC-3 and SGK1 expressions were assessed using Spearman rank correlation analysis, and overall survival curves were assessed using Kaplan–Meier analysis. The *P* values <0.05 were considered statistically significant.

## Results

### Olaparib exerted antitumor effect in STAD cell lines

**Fig. 1:**
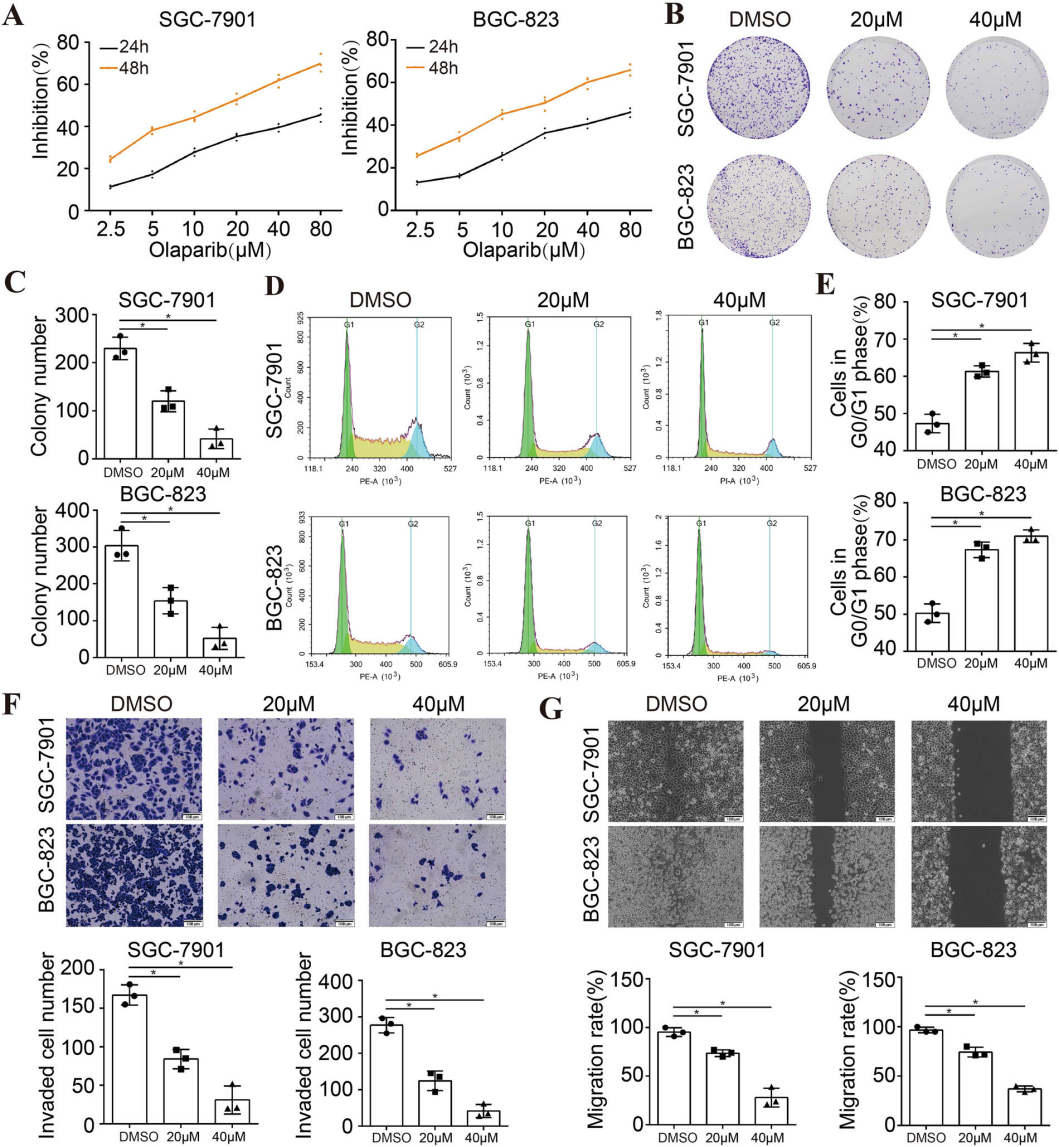
Olaparib exerted antitumor effect in STAD cell lines. **A** Olaparib inhibited the proliferation of STAD cells in a dose-dependent and time-dependent manner, with the IC_50_ value being ~20 μM in 48 h (*n* = 3). **B**, **C** Olaparib inhibited the clonogenicity of STAD cells in a dose-dependent manner (*n* = 3). **D**, **E** Olaparib arrested the cell cycle of STAD cells at the G0/G1 phase in a dose-dependent manner (*n* = 3). **F** Olaparib inhibited the invasion of STAD cells in a dose-dependent manner (*n* = 3). **G** Olaparib inhibited the migration of STAD cells in a dose-dependent manner (*n* = 3). *P < 0.05.

In this study, to verify whether olaparib exerted antitumor effect in STAD cells, the effect of olaparib on cell proliferation was first analyzed in two human STAD cell lines (SGC-7901 and BGC-823). In detail, different concentrations of olaparib (2.5, 5, 10, 20, 40, and 80 μM) were applied to STAD cells for 24 and 48 h. The results of the MTS assay showed that olaparib inhibited the proliferation of STAD cells in a dose-dependent and time-dependent manner, with the half-maximal inhibitory concentrations (IC_50_) value being ~20 μM in 48 h (19.03 ± 2.31 and 20.32 ± 1.64 μM for SGC-7901 and BGC-823 cells, respectively) (Fig. 1A). In the clone formation assay, we found that the formation number of cell clones was decreased in STAD cells treated with olaparib, and the decreased colony number was exhibited in a dose-dependent manner (Fig. 1B, C, S1a). Next, cell cycle analysis distribution was observed by flow cytometry. The results showed that the cell number at the G0/G1 phase was distinctly elevated in STAD cells treated with olaparib. Moreover, olaparib arrested the cell cycle in a dose-dependent manner (Figs. 1D, E, S1b). Transwell invasion assay was performed to determine the invasion ability of STAD cells. After 24 h, the invaded cells in the lower chambers were stained and counted under a light microscope. The data demonstrated that the invaded cell number was distinctly reduced in the cells treated with olaparib, and the reduction was exhibited in a concentration-dependent manner (Figs. 1F, S1c). To further evaluate the effect of olaparib on cell migration, scratch assay was conducted. Through the wound healing model, cell migration images were captured at 0 and 36 h. Consistent with the results above, the relative migration rate was inhibited by olaparib in a concentration-dependent manner (Figs. 1G, S1d). These findings proved that olaparib exerted antitumor effect in STAD cell lines.

### ClC-3/SGK1 regulatory axis was identified and validated in STAD cell lines

**Fig. 2:**
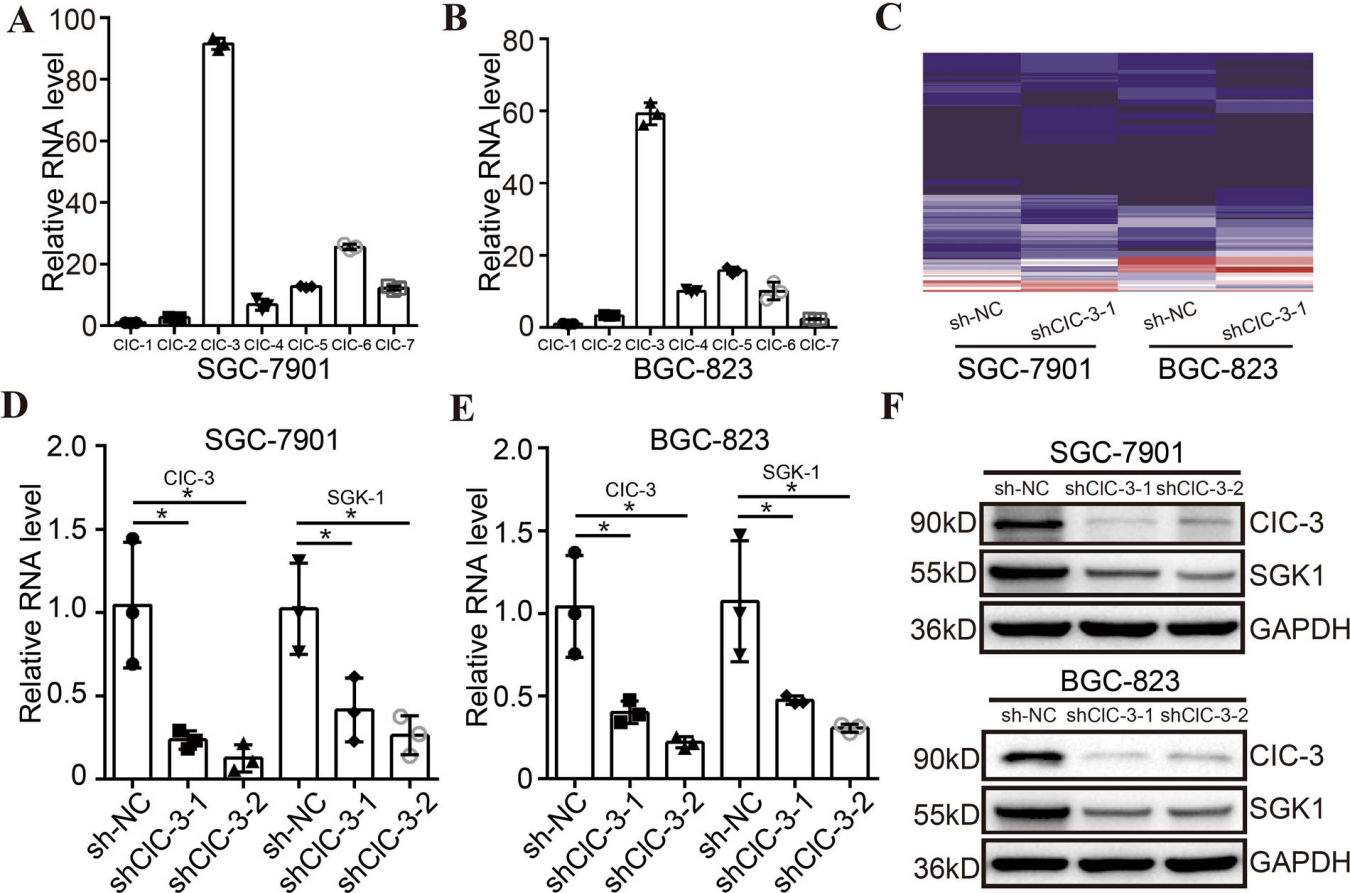
ClC-3/SGK1 regulatory axis was identified and validated in STAD cell lines. **A**, **B** The RNA level of ClC-3 was significantly abundant compared with other ClC chloride channel superfamily members (*n* = 3). **C** The heatmap of RNA sequencing revealed that 60 same genes were correspondingly down-regulated in the two ClC-3 KD cells, of which the SGK1 gene was the mostly down-regulated. **D**, **E** The RNA levels of ClC-3 and SGK1 were significantly reduced in the two ClC-3 KD cells (*n* = 3). **F** The protein levels of ClC-3 and SGK1 were significantly decreased in the two ClC-3 KD cells (*n* = 3). shNC negative control shRNA, shClC-3-1 ClC-3 knockdown shRNA-1, shClC-3-2 ClC-3 knockdown shRNA-2. *P < 0.05.

CIC chloride channel protein is a superfamily that mediates the transport of small molecule drugs across cell membranes. To further explore the action mechanism of olaparib in this study, the RNA levels of ClC chloride channel family members were first determined by qRT-PCR in STAD cells. The quantified results and representative amplification curves revealed that the RNA level of ClC-3 was significantly abundant compared with other ClC chloride channel superfamily members (Figs. 2A, B, S2a). By transfecting ClC-3 KD lentivirus, two stable ClC-3 KD STAD cell lines were established. To identify the downstream target gene of ClC-3, RNA sequencing for the gene expression profile was evaluated in two ClC-3 KD cells. The heatmap of RNA sequencing revealed that 60 same genes were correspondingly down-regulated in the two ClC-3 KD cells, of which the SGK1 gene was the mostly down-regulated (Fig. 2C, Table S2). Next, we validated whether the SGK1 gene was the downstream target of ClC-3. The quantified results and representative amplification curves showed that the RNA levels of ClC-3 and SGK1 were reduced in the two ClC-3 KD cells, which was identical to the RNA sequencing results (Figs. 2D, E, S2d, e, Table S2). In addition, at the protein level, the expression of ClC-3 and SGK1 was decreased in the two ClC-3 KD cells, confirming that SGK1 was regulated by ClC-3 at both the protein and RNA level in STAD cells (Figs. 2F, S2b, c). Altogether, ClC-3/SGK1 regulatory axis was identified and validated in STAD cell lines.

### Down-regulation of ClC-3/SGK1 axis attenuated olaparib-induced cell growth inhibition

**Fig. 3:**
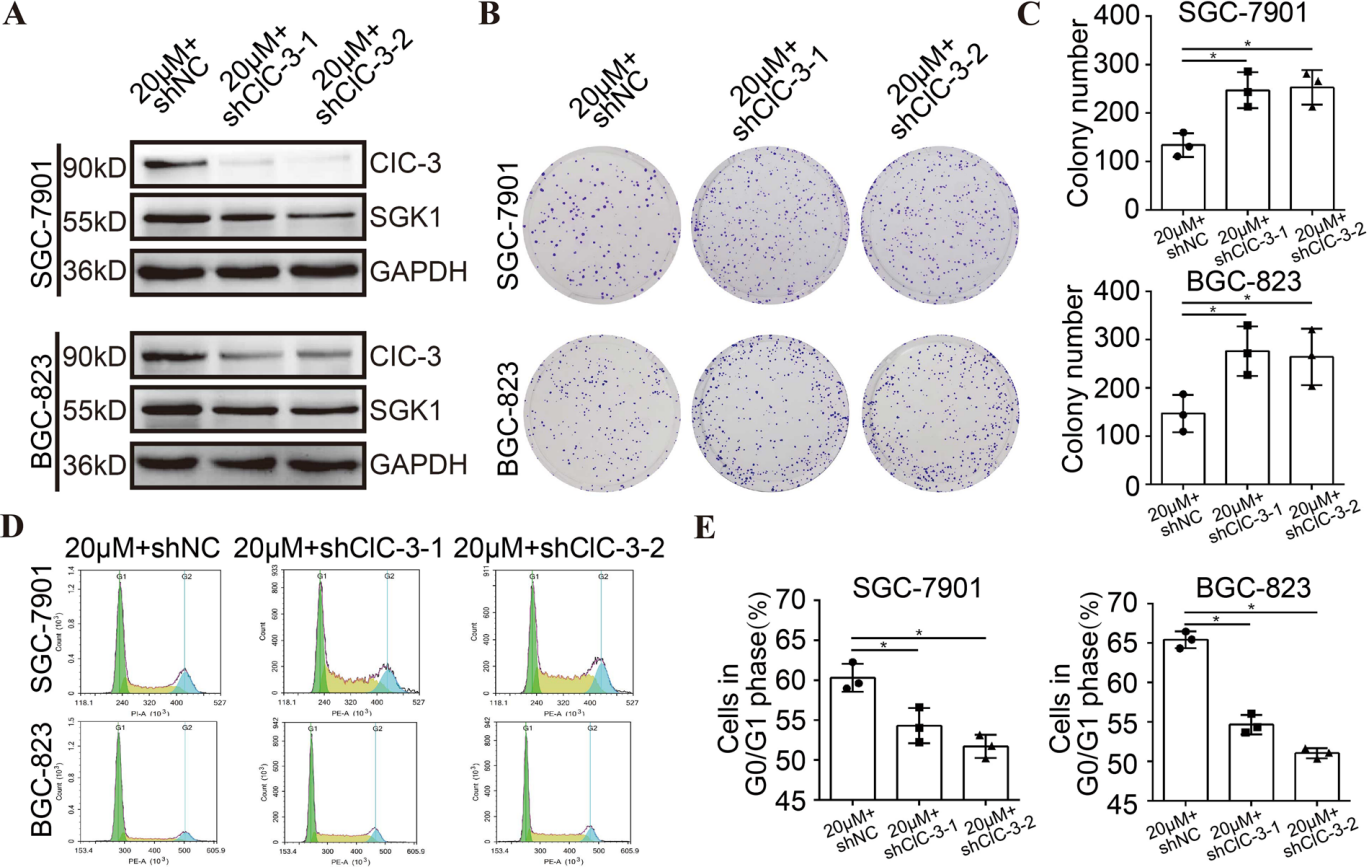
Down-regulation of ClC-3/SGK1 axis attenuated olaparib-induced cell growth inhibition. **A** The protein levels of ClC-3 and SGK1 were significantly decreased in the two ClC-3 KD cells treated with 20 μM olaparib (*n* = 3). shNC negative control shRNA, shClC-3-1 ClC-3 knockdown shRNA-1, shClC-3-2 ClC-3 knockdown shRNA-2. **B**, **C** Down-regulation of ClC-3/SGK1 axis attenuated olaparib-induced cell clonogenicity inhibition (*n* = 3). **D**, **E** Down-regulation of ClC-3/SGK1 axis attenuated olaparib-induced cell cycle arrest at the G0/G1 phase (*n* = 3). *P < 0.05.

To verify whether the ClC-3/SGK1 axis participated in the olaparib-induced antitumor effect, the two ClC-3 KD cells were treated with 20 μM olaparib. First, the WB results revealed that the protein levels of ClC-3 and SGK1 were decreased in the two ClC-3 KD cells treated with olaparib, which was consistent with the cells treated without olaparib, revealing that the ClC-3/SGK1 axis was down-regulated (Figs. 3A, S3a, b, c). Subsequently, the cell clonogenicity and cell cycle distribution were assessed in the two ClC-3 KD cells treated with olaparib. In the clone formation assay, the down-regulation of ClC-3/SGK1 axis attenuated olaparib-induced cell clonogenicity inhibition (Figs. 3B, C, S3d). Furthermore, in the cell cycle analysis by flow cytometry, the results showed that the down-regulation of ClC-3/SGK1 axis attenuated olaparib-induced cell cycle arrest at the G0/G1 phase (Figs. 3D, E, S3e). These findings proved that down-regulation of ClC-3/SGK1 axis attenuated olaparib-induced cell growth inhibition.

### Down-regulation of ClC-3/SGK1 axis attenuated olaparib-induced cell migration inhibition

**Fig. 4:**
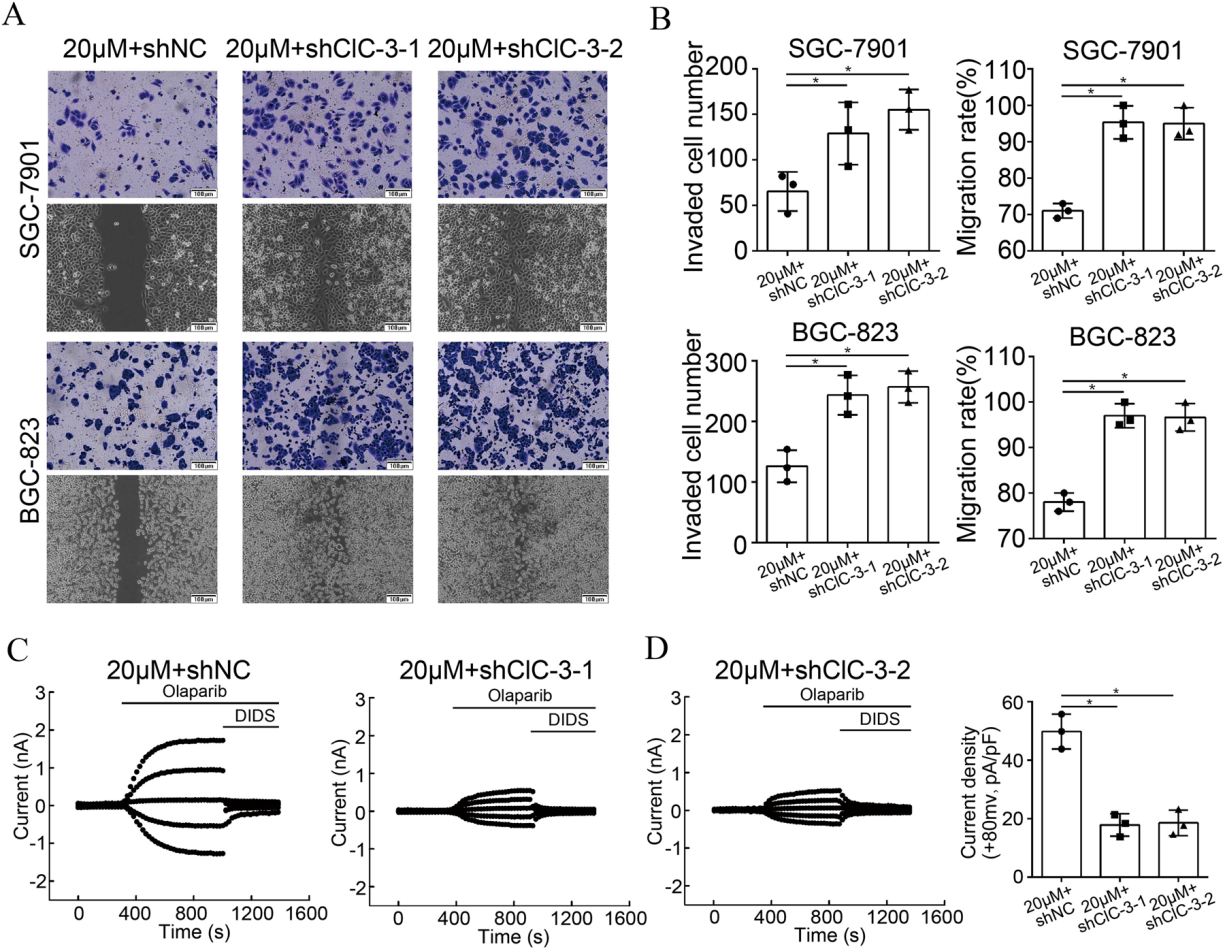
Down-regulation of ClC-3/SGK1 axis attenuated olaparib-induced cell migration inhibition. **A**, **B** Down-regulation of ClC-3/SGK1 axis attenuated olaparib-induced cell invasion and migration inhibition (*n* = 3). **C** Typical time courses of the chloride current recorded by whole-cell patch clamp. The results indicated that the chloride current activated by olaparib was decreased in ClC-3 KD SGC-7901 cells (*n* = 3). **D** Current density analysis indicated that the chloride current density activated by olaparib was decreased in ClC-3 KD SGC-7901 cells (*n* = 3). *P < 0.05.

To clarify whether the ClC-3/SGK1 axis participated in olaparib-induced migration, transwell assay and scratch assay were conducted in the two ClC-3 KD cells treated with olaparib. We found that the down-regulation of ClC-3/SGK1 axis attenuated olaparib-induced cell invasion and migration inhibition (Figs. 4A, B, S4a, b). ClC-3 chloride channel has been reported to mediate chloride current formation in the membrane. To further confirm the crucial role of the ClC-3 chloride channel in the olaparib-induced antitumor effect, the whole-cell patch clamp technique was used to assess the effect of olaparib on chloride current formation in the membrane. In this study, whole-cell chloride current under isotonic (300 mosmol/l) conditions was recorded in ClC-3 NC and ClC-3 KD SGC-7901 cells. DIDS, a commonly used chloride channel blocker, was used to ensure the accuracy of the recorded chlorine current. First, the instantaneous basal chloride current results indicated that there was no significant difference between ClC-3 NC and ClC-3 KD cells before stimulation (Fig. S4c). Next, the whole and instantaneous recorded images showed that the chloride current was activated by olaparib, and the chloride current activated by olaparib was decreased in ClC-3 KD cells (Figs. 4C, S4d). As expected, the current density analysis indicated that the chloride current density activated by olaparib was also decreased in ClC-3 KD cells (Fig. 4D). These findings proved that down-regulation of ClC-3/SGK1 axis attenuated olaparib-induced cell migration inhibition, as well as olaparib-activated chloride current.

### Up-regulation of ClC-3/SGK1 axis enhanced olaparib-induced cell growth inhibition

**Fig. 5:**
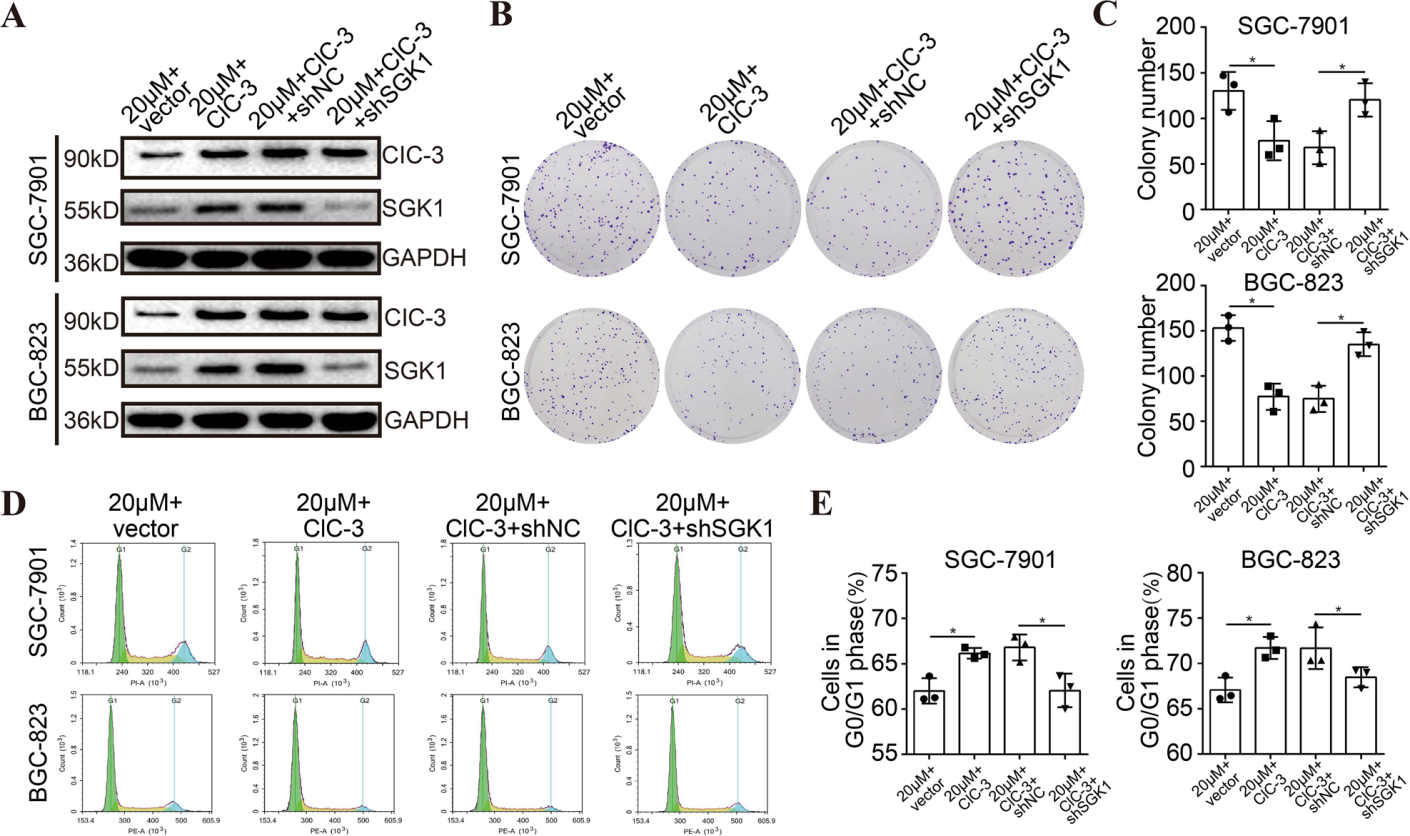
Up-regulation of ClC-3/SGK1 axis enhanced olaparib-induced cell growth inhibition. **A** The protein levels of ClC-3 and SGK1 were significantly increased in the two ClC-3 OV cells treated with 20 μM olaparib (*n* = 3). vector empty vector-transfected cells, ClC-3 ClC-3 overexpression cells, ClC-3+shNC ClC-3 overexpression and SGK1 negative control cells, ClC-3+ shSGK1 ClC-3 overexpression and SGK1 knockdown cells. **B**, **C** Up-regulation of ClC-3/SGK1 axis enhanced olaparib-induced cell clonogenicity inhibition, and the enhancement effect could be attenuated by SGK1 KD (*n* = 3). **D**, **E** Up-regulation of ClC-3/SGK1 axis enhanced olaparib-induced cell cycle arrest at the G0/G1 phase, and the enhancement effect could be attenuated by SGK1 KD (*n* = 3). * *P* < 0.05.

To further explore the molecular mechanisms underlying the antitumor effect of olaparib, two stable ClC-3 OV STAD cell lines were established. By transfecting SGK1 KD lentivirus, stable ClC-3 OV + SGK1 KD cell lines were also established. WB results revealed that the protein levels of ClC-3 and SGK1 were increased in the two ClC-3 OV cells treated with olaparib, revealing that the ClC-3/SGK1 axis was up-regulated (Figs. 5A, S5a, b, c). Subsequently, the cell clonogenicity and cell cycle distribution were assessed in the two ClC-3 OV cells treated with olaparib. In the clone formation assay, the up-regulation of ClC-3/SGK1 axis enhanced olaparib-induced cell clonogenicity inhibition, but the enhancement effect could be attenuated by SGK1 KD (Figs. 5B, C, S5d). Furthermore, in the cell cycle analysis by flow cytometry, the results showed that the up-regulation of ClC-3/SGK1 axis enhanced olaparib-induced cell cycle arrest at the G0/G1 phase, but the enhancement effect could be attenuated by SGK1 KD (Figs. 5D, E, S5e). These findings proved that up-regulation of ClC-3/SGK1 axis enhanced olaparib-induced cell growth inhibition. Moreover, both ClC-3 and SGK1 were crucial in olaparib-induced antitumor effect.

### Up-regulation of ClC-3/SGK1 axis enhanced olaparib-induced cell migration inhibition

**Fig. 6:**
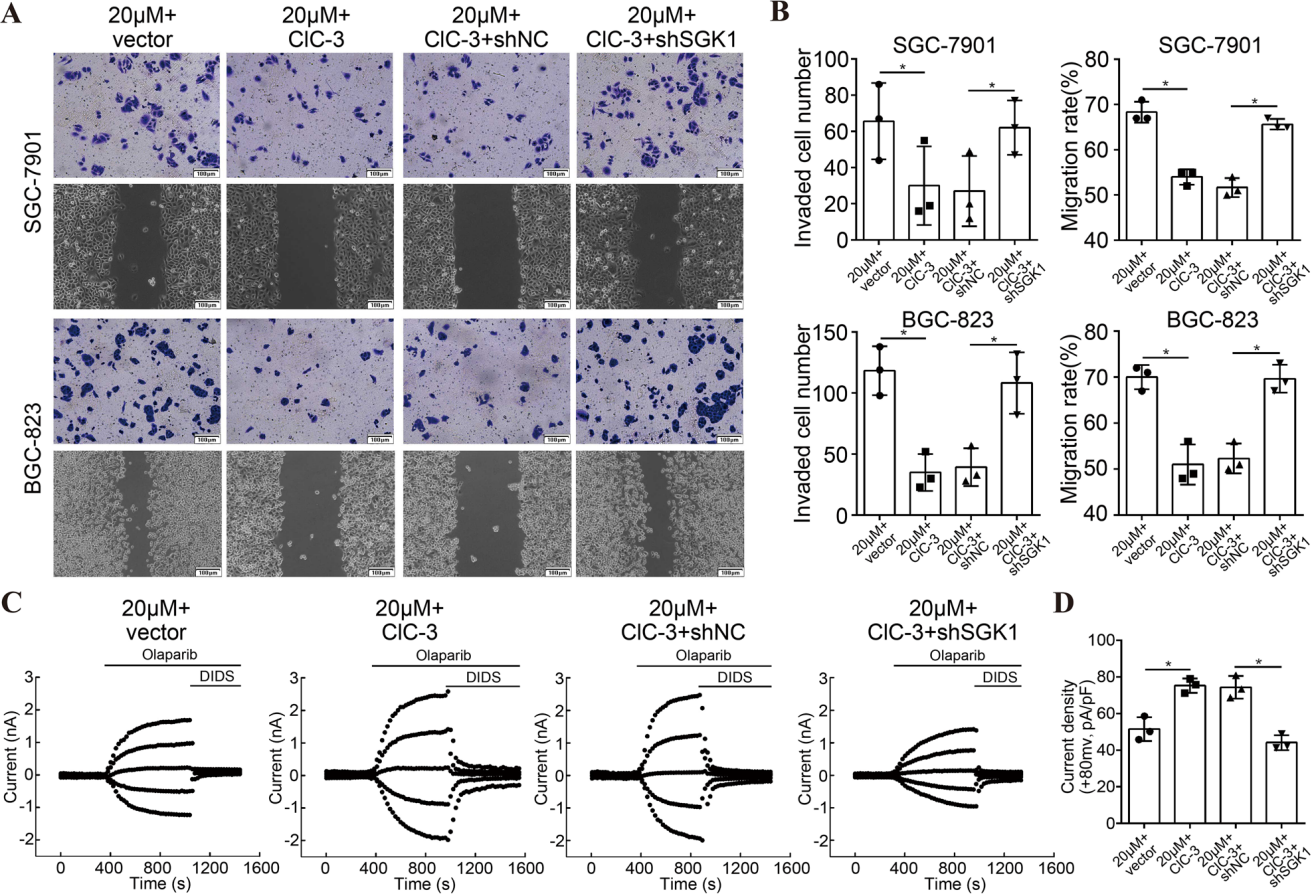
Up-regulation of ClC-3/SGK1 axis enhanced olaparib-induced cell migration inhibition. **A**, **B** Up-regulation of ClC-3/SGK1 axis enhanced olaparib-induced cell invasion and migration inhibition, and the enhancement effect could be attenuated by SGK1 KD (*n* = 3). **C** Typical time courses of the chloride current recorded by whole-cell patch clamp. The results indicated that the chloride current activated by olaparib was increased in ClC-3 OV SGC-7901 cells, and the increase effect could be attenuated by SGK1 KD (*n* = 3). **D** Current density analysis indicated that the chloride current density activated by olaparib was increased in ClC-3 OV SGC-7901 cells, and the increase effect could be attenuated by SGK1 KD (*n* = 3). *P < 0.05.

To clarify whether the ClC-3/SGK1 axis participated in olaparib-induced migration, transwell assay and scratch assay were conducted in the two ClC-3 OV cells treated with olaparib. We found that the up-regulation of ClC-3/SGK1 axis enhanced olaparib-induced cell invasion and migration inhibition, but the enhancement effect could be attenuated by SGK1 KD (Figs. 6A, B, S6a, b). Similarly, the whole-cell patch clamp technique was used to assess the effect of olaparib on the chloride current in the membrane. First, the instantaneous basal chloride current results indicated that there was no significant difference among ClC-3 vector, ClC-3 OV, ClC-3 OV + SGK1 NC, and ClC-3 OV + SGK1 KD cells before stimulation (Fig. S6c). Next, the whole and instantaneous recorded images showed that the chloride current activated by olaparib was increased in ClC-3 OV cells, and the increase effect could be attenuated by SGK1 KD (Fig. 6C, S6d). As expected, the chloride current density activated by olaparib was increased in ClC-3 OV cells, and the increase effect could be attenuated by SGK1 KD (Fig. 6D). These findings proved that up-regulation of ClC-3/SGK1 axis enhanced olaparib-induced cell migration inhibition, as well as olaparib-activated chloride current. Importantly, our data confirmed the crucial roles of ClC-3 and SGK1 in olaparib-induced antitumor effect.

### ClC-3 and SGK1 were positively correlated and highly expressed in human STAD tissues

**Fig. 7:**
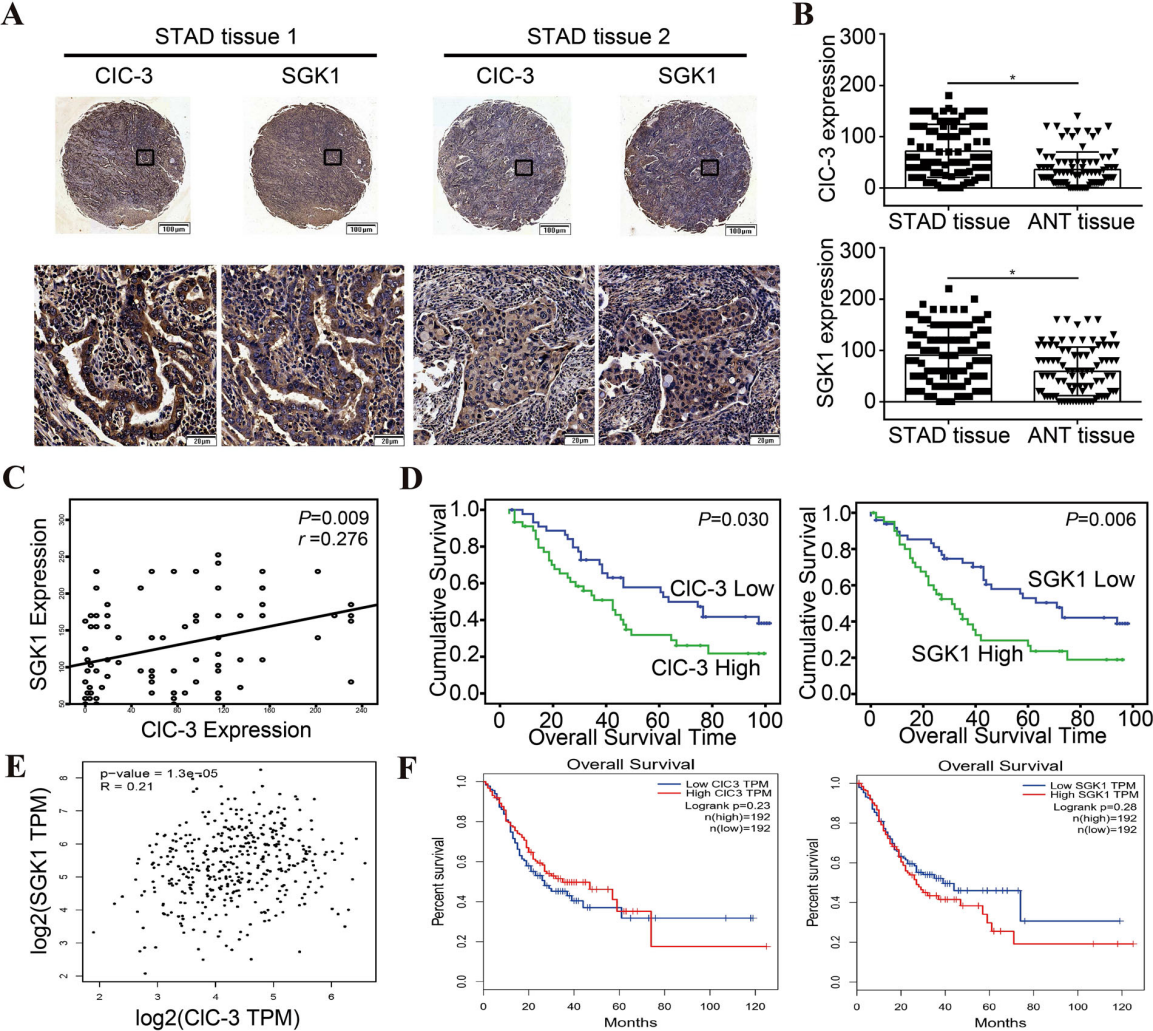
ClC-3 and SGK1 were positively correlated and highly expressed in human STAD tissues. **A** Representative images of ClC-3 and SGK1 protein expression in human STAD tissues. **B** The protein expression of ClC-3 and SGK1 in 90 STAD tissues was higher than that in ANTs. **C** The protein expression of ClC-3 and SGK1 in 90 STAD tissues was positively correlated. **D** High protein expression of ClC-3 was associated with poor survival rate in STAD patients (left), and high protein expression of SGK1 was also associated with poor survival rate in STAD patients (right). **E** The transcript per million (TPM) expression of ClC-3 and SGK1 was positively correlated in STAD tissues. **F** High TPM expression of ClC-3 tended to predict poor survival rate in STAD patients (left), and high TPM expression of SGK1 tended to predict poor survival rate in STAD patients (right). *P < 0.05.

Given the pivotal role of the ClC-3/SGK1 regulatory axis in olaparib-induced antitumor effect, we then evaluated the clinical significance of the ClC-3/SGK1 axis in STAD patients. Through IHC staining of 90 human STAD tissues, we found that the protein expression of ClC-3 and SGK1 in 90 STAD tissues was higher than that in adjacent normal tissues (ANTs) (Figs. 7A, B, S7a). Furthermore, the representative IHC images showed that ClC-3 and SGK1 were mainly localized in the cytoplasm (Fig. 7A). As shown in the images, the STAD tissue with high ClC-3 expression tended to have a high level of SGK1, so the correlation analysis between ClC-3 and SGK1 was further investigated. The Spearman rank correlation analysis showed that the protein expression of ClC-3 and SGK1 was positively correlated (*r* = 0.276, *P* = 0.009) (Fig. 7C). Next, the Kaplan–Meier survival analysis revealed that high protein expression of ClC-3 was associated with poor survival rate in STAD patients (*P* = 0.030). Meanwhile, high protein expression of SGK1 was also associated with poor survival rate in STAD patients (*P* = 0.006) (Fig. 7D). To further investigate whether the expression of ClC-3 and SGK1 was correlated at the RNA level, the transcript per million (TPM) expression of ClC-3 and SGK1 was obtained from the gene expression profiling interactive analysis (GEPIA) web datasets. Similarly, the TPM expression of ClC-3 and SGK1 was positively correlated in STAD tissues (*r* = 0.21, *P* < 0.001) (Fig. 7E). Overall survival results showed that high TPM expression of ClC-3 tended to predict poor survival rate in STAD patients, as well as high TPM expression of SGK1 (Fig. 7F). Finally, the correlations between ClC-3/SGK1 and their downstream molecules in STAD tissues were obtained from the GEPIA datasets. The data revealed that the TPM expression of ClC-3 and AKT1 was positively correlated (*r* = 0.29, *P* < 0.001) (Fig. S7b), and the TPM expression of SGK1 and AKT1 was positively correlated (*r* = 0.17, *P* < 0.001) (Fig. S7c). Additionally, we found that the TPM expression of SGK1 and Cyclin D1/MMP2/MMP9 was also positively correlated in STAD tissues (*r* = 0.15, *P* < 0.01; *r* = 0.26, *P* < 0.001; *r* = 0.23, *P* < 0.001, respectively) (Fig. S7d–f). These findings proved that ClC-3 and SGK1 were positively correlated at both the protein and RNA level in human STAD tissues. Importantly, high expression of both ClC-3 and SGK1 was associated with poor survival rate in STAD patients, indicating that the ClC-3/SGK1 axis might be a prognostic marker of overall survival in STAD patients.

### Olaparib inhibited the downstream PI3K/AKT pathway of ClC-3/SGK1 axis and exerted antitumor effect in vivo

**Fig. 8:**
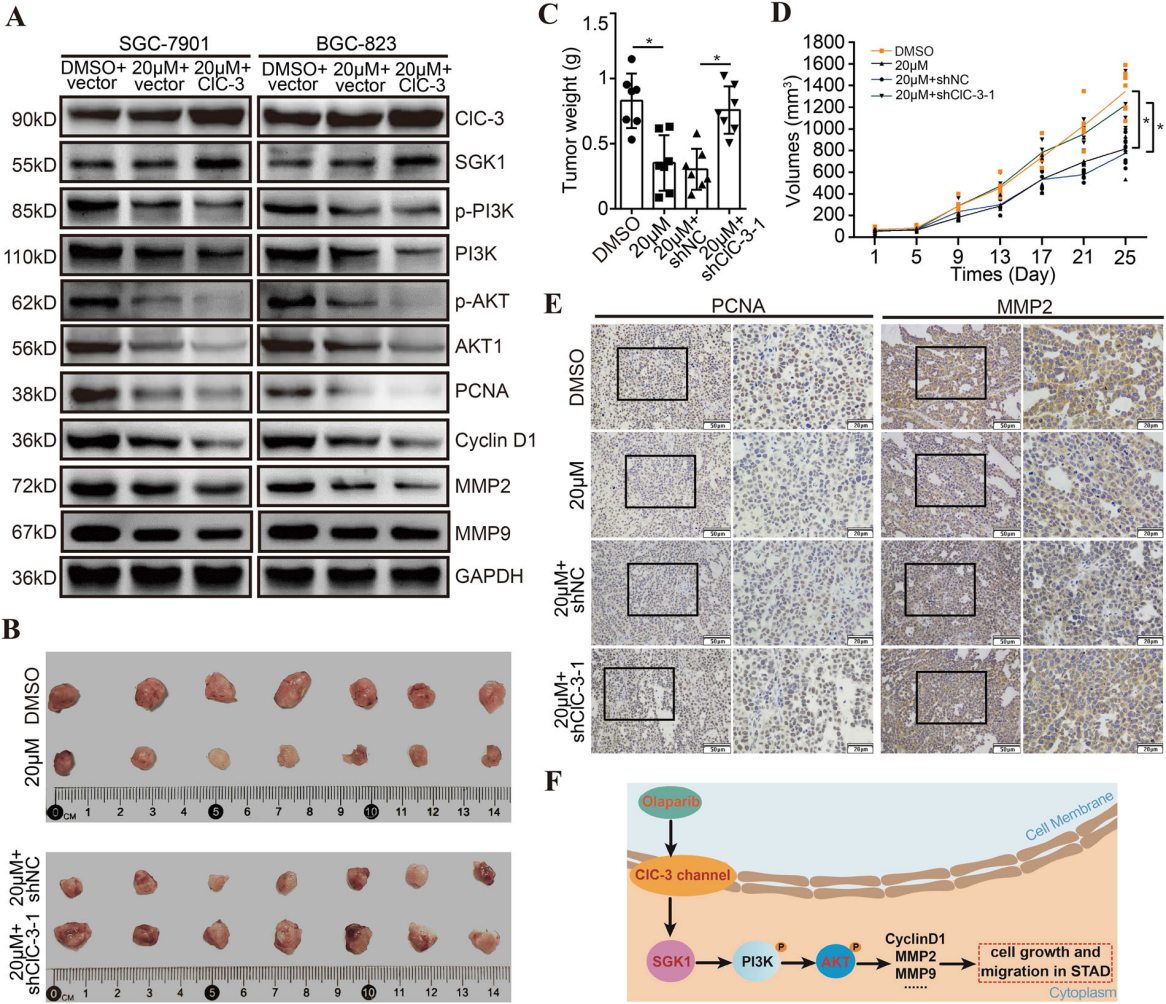
Olaparib inhibited the downstream PI3K/AKT pathway of ClC-3/SGK1 axis and exerted antitumor effect in vivo. **A** Olaparib inhibited the PI3K/AKT pathway in STAD cells, and up-regulation of ClC-3/SGK1 axis enhanced olaparib-induced PI3K/AKT pathway inhibition (*n* = 3). vector, empty vector-transfected cells; ClC-3 ClC-3 overexpression cells. **B** Nude mice were subcutaneously injected with SGC-7901 cells, and representative images of STAD tumor xenografts were exhibited (*n* = 7 mice/group). **C** Down-regulation of ClC-3 attenuated olaparib-induced tumor weight reduction. **D** Down-regulation of ClC-3 attenuated olaparib-induced tumor volume decrease. **E** The expression of PCNA and MMP2 in tumor tissues was detected by IHC staining. **F** The proposed mechanism model of olaparib in STAD growth and migration. *P < 0.05.

Based on the results above, we hypothesized that the downstream PI3K/AKT pathway of ClC-3/SGK1 axis was involved in olaparib-induced antitumor effect. In the ClC-3 vector cells treated with olaparib, the WB results showed that the protein levels of p-PI3K, PI3K, p-AKT, AKT1, PCNA, CyclinD1, MMP2, and MMP9 were decreased. Furthermore, in the ClC-3 OV cells treated with olaparib, we found that the protein levels of p-PI3K, PI3K, p-AKT, AKT1, PCNA, CyclinD1, MMP2, and MMP9 were more obviously inhibited, indicating that the up-regulation of ClC-3/SGK1 axis enhanced olaparib-induced PI3K/AKT pathway inhibition (Figs. 8A, S8a–c). Next, the olaparib-induced antitumor effect was evaluated in vivo. ClC-3 NC and ClC-3 KD SGC-7901 cells were subcutaneously injected into the left flank of nude mice, and tumors developed at the injection sites after one week. Tumor volumes were measured every 5 days, and the tumor xenografts were weighed and processed for IHC staining after ~4 weeks. Consistent with the in vitro results, the representative tumor images showed that down-regulation of ClC-3 attenuated olaparib-induced tumor growth inhibition (Fig. 8B). In detail, down-regulation of ClC-3 attenuated olaparib-induced tumor weight reduction and volume decrease (Figs. 8C, D, S8d, e). Finally, the proliferation marker PCNA and the invasion marker MMP2 were detected by IHC staining in tumor xenografts. The staining images showed that down-regulation of ClC-3 attenuated olaparib-induced PCNA/MMP2 expression inhibition (Fig. 8E). These findings proved that olaparib inhibited the downstream PI3K/AKT pathway of ClC-3/SGK1 axis and exerted antitumor effect in vivo.

## Discussion

Currently, molecular-targeted therapy has been revolutionized and applied in several different types of cancers. However, the molecular-targeted therapeutic strategy of digestive system tumors still continues to be a serious challenge in the clinic. Especially in patients with STAD, only a few available targeted therapeutic drugs are considered to be effective^21–23^. Therefore, there is a need to explore more targeted drugs for STAD for therapeutic purposes. Recently, targeting poly-ADP-ribose polymerase (PARP) has been identified as a promising option against BRCA-defective cancer. For instance, the PARP inhibitor olaparib has been approved to treat patients with BRCA-defective ovarian or breast cancer. However, there is no current evidence that BRCA mutations are widely existed in STAD patients. Meanwhile, the effect of olaparib on STAD cells and its underlying mechanisms remain uninvestigated, which largely restricts the use of PARP inhibitors in STAD patients, as well as other tumors without BRCA gene mutations. For the first time, we found that olaparib exerted antitumor effect in vitro and in vivo. Importantly, our findings illustrated that the ClC-3/SGK1 regulatory axis enhanced the olaparib-induced antitumor effect in human STAD. This work revealed that up-regulation of the ClC-3/SGK1 regulatory axis might provide promising therapeutic potential for the clinical application of olaparib in STAD treatment.

In previous reports, most of the studies have focused on the effect of olaparib on DNA damage and apoptosis in malignant tumors. Here, our results showed that olaparib exerted antitumor effect in STAD cells, with a novel function in regulating cell growth and cell migration. In detail, the olaparib-induced cell proliferation inhibition occurred in a dose-dependent and time-dependent manner, which was in accordance with Lin’s findings in MKN45 and AGS cells^24^. Owing to the IC_50_ value of olaparib in STAD cells, 20 and 40 μM olaparib were used in the subsequent studies. As expected, olaparib obviously inhibited cell clonogenicity in a dose-dependent manner, with the cell cycle mostly arrested at the G0/G1 phase. It is known that blocking cell cycle progression can inhibit cell proliferation. Recent study has shown that the cell proliferation can be affected by the formation of cell cycle-dependent complex CDK6/CCND1^25^. Clinically, the treatment of cell cycle-specific agents is based on arresting tumor cells at the G1/S border^26,27^. Therefore, our results indicated that olaparib might attenuate cell growth through regulation of the cell cycle. STAD cells are characterized by a high risk of metastasis^28^. In this study, the inhibitory activity of olaparib on cell invasion and migration was also verified in a dose-dependent manner. Cell migration process requires many factors, such as the cytoskeleton, cell–matrix adhesion and cell volume regulation^29^. When the cell proliferation is inhibited, a broad range of cellular characteristics will be orchestrated, including the matrix environmental and cell mechanical properties, which is of importance to the cell migration and contributes to the invasive phenotype of cancer cells^30–32^. Our previous study has showed that the cell cycle also plays an important role in controlling cell migration and cell volume^33,34^. Thus, the olaparib-induced cell growth inhibition and migration inhibition were parallel and interrelated. Taken together, these findings confirmed that olaparib exerted antitumor effect in STAD cell lines. However, remarkable inhibitions of the cell cycle and invasion were rarely reported in other olaparib-related experiments, prompting us to further examine the underlying molecular mechanism of olaparib in STAD cells.

The voltage-gated chloride channel superfamily is a class of transmembrane protein that is essential for multiple physiological processes and appears to be involved in the transport and susceptibility of anticancer drugs^14^. Members of this superfamily can be mainly classified from ClC-1 to ClC-7, of which ClC-3 is the research hotspot in cancer therapy^15^. However, the expression and function of ClC-3 in STAD are still unclear. In this study, we found that the RNA level of ClC-3 was significantly abundant compared with other ClC chloride channel superfamily members, which was coincided with the results in nasopharyngeal carcinoma cells, indicating that ClC-3 might play a crucial role in STAD progression. Previous studies have shown that opening of the ClC-3 chloride channel contributed to increased drug sensitivity^17,35^, so we hypothesized that the ClC-3 pathway might be involved in the olaparib-induced antitumor effect. As a serine/threonine protein kinase, SGK1 is involved in the regulation of processes such as stress response, material transport, gene transcription, and neuronal excitability^18,36^. Recently, it is reported that SGK1 is up-regulated in a variety of tumor types, and involved in the growth, survival, autophagy, and drug resistance of tumor cells^37,38^. Through RNA sequencing, SGK1 was identified as the main downstream target gene of ClC-3 in STAD cells. In the ClC-3 KD cells, SGK1 was regulated by ClC-3 at both the protein and RNA level, validating that the ClC-3/SGK1 regulatory axis was existed in STAD cells. Studies have shown that SGK1 can be regulated by WNK1, mTORC1, and miR-576-3p^39–41^. But in this work, we discovered that the ion channel protein ClC-3 was also a regulatory factor of SGK1. We supposed that ClC-3 should not be the only regulator of SGK1, and the other molecular regulators would be explored in the future work. Based on the work above, we speculated that the ClC-3/SGK1 regulatory axis might be involved in the olaparib-induced antitumor effect.

The potential antitumor mechanism of olaparib in STAD cells remains elusive. In our study, accumulating evidence pointed to the key roles of the ClC-3/SGK1 regulatory axis. In the ClC-3 KD cells, we found that down-regulation of ClC-3/SGK1 axis attenuated olaparib-induced cell growth inhibition. Conversely, in the ClC-3 OV cells, the results demonstrated that up-regulation of ClC-3/SGK1 axis enhanced olaparib-induced cell growth inhibition, and the enhancement effect could be attenuated by SGK1 KD. Much the same was observed in the olaparib-induced cell migration inhibition. Based on the results above, we proved that olaparib inhibited cell growth and migration via ClC-3 chloride channel and the following ClC-3/SGK1 regulatory axis in STAD cells, and both ClC-3 and SGK1 were indispensable in olaparib-induced antitumor effect. Meanwhile, in ClC-3 KD and ClC-3 OV cells treated with olaparib, the expression changes of ClC-3 and SGK1 further validated the existence of the ClC-3/SGK1 regulatory axis in STAD cells. Previous mechanistic studies of olaparib mostly focused on the regulation of the prevention of base-excision DNA repair, which resulted in tumor cell death^42,43^. This work provided a new mechanism by which olaparib inhibited the cell growth and migration in STAD cells.

Ion channels are ubiquitous messengers in cancer, serving as a signaling molecule for a variety of cellular processes such as control of the cell cycle, apoptosis, and migration. Various signaling pathways are closely related with the ion channels, such as NF-κB, Wnt/β-catenin, PI3K/Akt, and MAPK. The ClC-3 chloride channel is thought to act as an anion channel protein, which is involved in controlling membrane conductance, chloride ion signaling, and PI3K/Akt signaling in proliferating cells^13^. Parameters such as the membrane chloride current, intracellular chloride ion concentration, cytosolic pH, and cell volume are governed by the ClC-3 chloride channel^14,15^. Studies have shown that the swelling-induced chloride current contributes to the proliferation and invasion of human glioma cells^44^. Moreover, in prostate cancer epithelial cells, Bcl-2-induced chloride current is mediated by ClC-3 and is associated with cell survival and cell apoptosis^45^. Especially in nasopharyngeal carcinoma cells, activation of the ClC-3 chloride current by small molecule drug is essential for cell proliferation and cell migration progression^17,46,47^. To confirm the role of ClC-3 in the olaparib-induced antitumor effect, the chloride current was recorded in ClC-3 KD and ClC-3 OV cells. First, the basal chloride current results showed that there was no significant difference among all groups, indicating that the basal chloride current levels were basically identical. Then the olaparib-activated chloride current levels of all groups were recorded. The results revealed that the chloride current activated by olaparib was decreased in ClC-3 KD cells. By contrast, the chloride current activated by olaparib was increased in ClC-3 OV cells, and the increase effect could be attenuated by SGK1 KD. Similarly, the chloride current density activated by olaparib presented the same variations. These results indicated that the ClC-3/SGK1 regulatory axis was a positive regulator of the chloride current in the membrane, and the antitumor effect of olaparib was related to the level of the activated chloride current in STAD cells. In detail, when the chloride current activated by olaparib was increased, the antitumor effect of olaparib was further promoted, suggesting that the ClC-3/SGK1 regulatory axis enhanced the sensitivity of cells to olaparib. The chloride current activation in the membrane can be influenced by multiple factors^14^. Recently, SGK1 has been implicated to modulate the activities of ion channels^48^, such as sodium channel (SCNN1A), potassium channel (KCNJ1), epithelial calcium channels (TRPV5), and chloride channel (BSND and CFTR). In our results, SGK1 KD reduced the olaparib-activated chloride current, suggesting that both ClC-3 chloride channel and SGK1 were indispensable to the chloride current activation. We speculated that SGK1 might regulate chloride current activation by interacting with ClC-3 or other chloride channels (BSND and CFTR), collectively modulating the chloride current level and current density. But it was still unclear about the specific mechanism of SGK1 in the chloride current activation, which provided us a new research direction for the future studies. Based on the data above, we confirmed that olaparib exerted antitumor effect via the ClC-3/SGK1 regulatory axis in STAD cell lines.

Previous studies have reported that ClC-3 is overexpressed in nasopharyngeal carcinoma and glioma^20,49^. As a protein kinase, SGK1 also has elevated expression in multiple tumor types, including prostate cancer, endometrial cancer and colorectal cancer^50–52^. To provide valuable clinical information of the ClC-3/SGK1 regulatory axis, the expression of ClC-3 and SGK1 was examined in STAD patients. At the protein level, the IHC results showed that ClC-3 and SGK1 were highly expressed. Moreover, the IHC staining of ClC-3 and SGK1 presented the same variation trend, which was consistent with their positive correlation observed in STAD tissues. The survival analysis indicated that high expression of both ClC-3 and SGK1 was associated with poor survival rate in STAD patients, suggesting that ClC-3 and SGK1 might be tumor-promoting factors in STAD development. At the RNA level, the transcript per million (TPM) expression of ClC-3 and SGK1 was also positively related and tended to predict the poor survival rate. Therefore, we speculated that double detection of ClC-3 and SGK1 could provide precise information for predicting the prognosis of STAD patients. It has been reported that SGK1 is highly homologous to the AKT kinase family^53^. Hence, the correlation analysis between AKT1 and SGK1 was explored, along with the correlation analysis between AKT1 and ClC-3. Our results revealed that AKT1 was positively correlated with ClC-3/SGK1 in STAD tissues, revealing that the correlation between ClC-3/SGK1 axis and AKT1 might be involved in STAD progression.

AKT1 belongs to the family of serine/threonine protein kinases (AKT1, AKT2, and AKT3) known as AKT kinases. Full activation of AKT is regulated by multiple components of the well-known PI3K/AKT pathway, which plays a key role in regulating cell survival and tumor formation^54^. Moreover, the PI3K/AKT signaling pathway has been reported to be the downstream of ClC-3^55^. So we hypothesized that the PI3K/AKT signaling pathway might be involved in the olaparib-induced antitumor effect. As shown in our findings, the levels of p-PI3K, PI3K, p-AKT, and AKT1 were inhibited by olaparib, and this inhibition was amplified by up-regulation of ClC-3/SGK1 axis. These results demonstrated that olaparib inhibited the PI3K/AKT pathway in STAD cells, and up-regulation of ClC-3/SGK1 axis enhanced olaparib-induced PI3K/AKT pathway inhibition. It is clear that PCNA, CyclinD1, MMP2, and MMP9 are downstream effectors of PI3K/AKT pathway involved in tumor invasion and growth^56–58^. In our study, the protein levels of these effectors presented the same variation trend. Moreover, the TPM expression of Cyclin D1/MMP2/MMP9 and SGK1 was positively correlated, revealing that the PI3K/AKT pathway and SGK1 axis were indeed interrelated. In the future, the molecular mechanism between the PI3K/AKT pathway and ClC-3/SGK1 regulatory axis would be further elucidated. In general, these data indicated that olaparib inhibited the downstream PI3K/AKT pathway of ClC-3/SGK1 axis and the pathway effectors.

The olaparib-induced antitumor effect was also investigated in mouse xenograft models. The results were consistent with our in vitro data and showed that down-regulation of ClC-3 attenuated olaparib-induced antitumor effect in vivo, along with the olaparib-induced PCNA/MMP2 expression inhibition. Therefore, the above data showed that olaparib also exerted antitumor effect in vivo. Here, we propose a model for the mechanism of olaparib in STAD growth and migration (Fig. 8F). First, ClC-3/SGK1 regulatory axis is existed in STAD cells, and olaparib inhibits the cell growth and migration via the ClC-3/SGK1 regulatory axis. Second, olaparib inhibits the downstream PI3K/AKT pathway of ClC-3/SGK1 axis and the pathway effectors, thus exerting antitumor effect in human STAD. In summary, this work illustrates that olaparib exerts antitumor effect in human STAD, and ClC-3/SGK1 regulatory axis enhances the olaparib-induced antitumor effect. Up-regulation of the ClC-3/SGK1 axis may provide promising therapeutic potential for the clinical application of olaparib in STAD treatment.

## Supplementary information

Figure S1

Figure S2

Figure S3

Figure S4

Figure S5

Figure S6

Figure S7

Figure S8

Table S1

Table S2

Supplementary Figure Legends

Supplementary Table Legends

## Data Availability

The datasets used and/or analyzed during the current study are available from the corresponding author on reasonable request.
