# Supplementary figures and images for: ClC-3/SGK1 regulatory axis enhances the olaparib-induced antitumor effect in human stomach adenocarcinoma
Source: Cell Death Dis. 2020 Oct 22;11(10):898. doi: 10.1038/s41419-020-03107-3 (PMC7583252; doi:10.1038/s41419-020-03107-3)

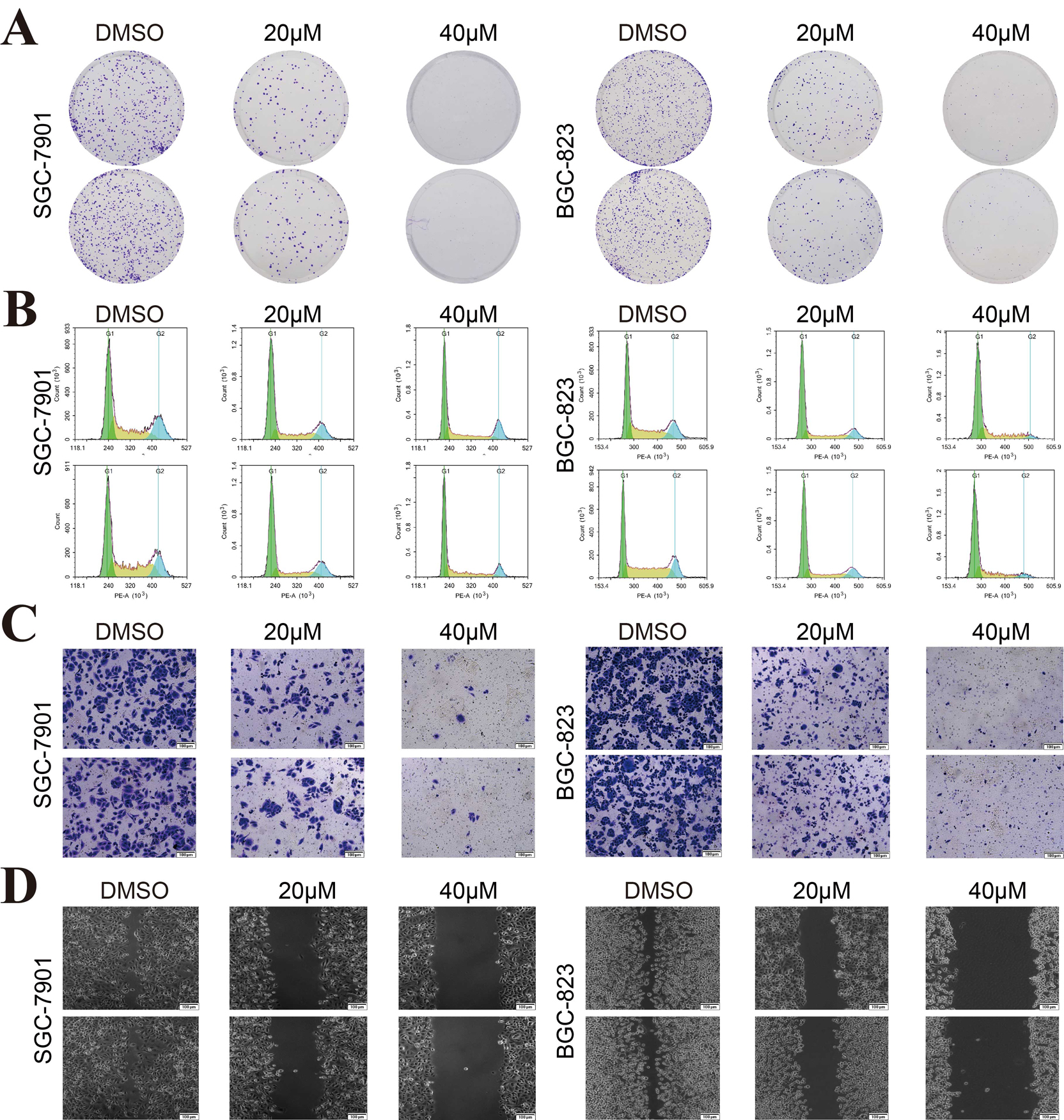

Supplement: Supplementary file 1 — Figure S1 [file 41419_2020_3107_MOESM1_ESM.jpg]

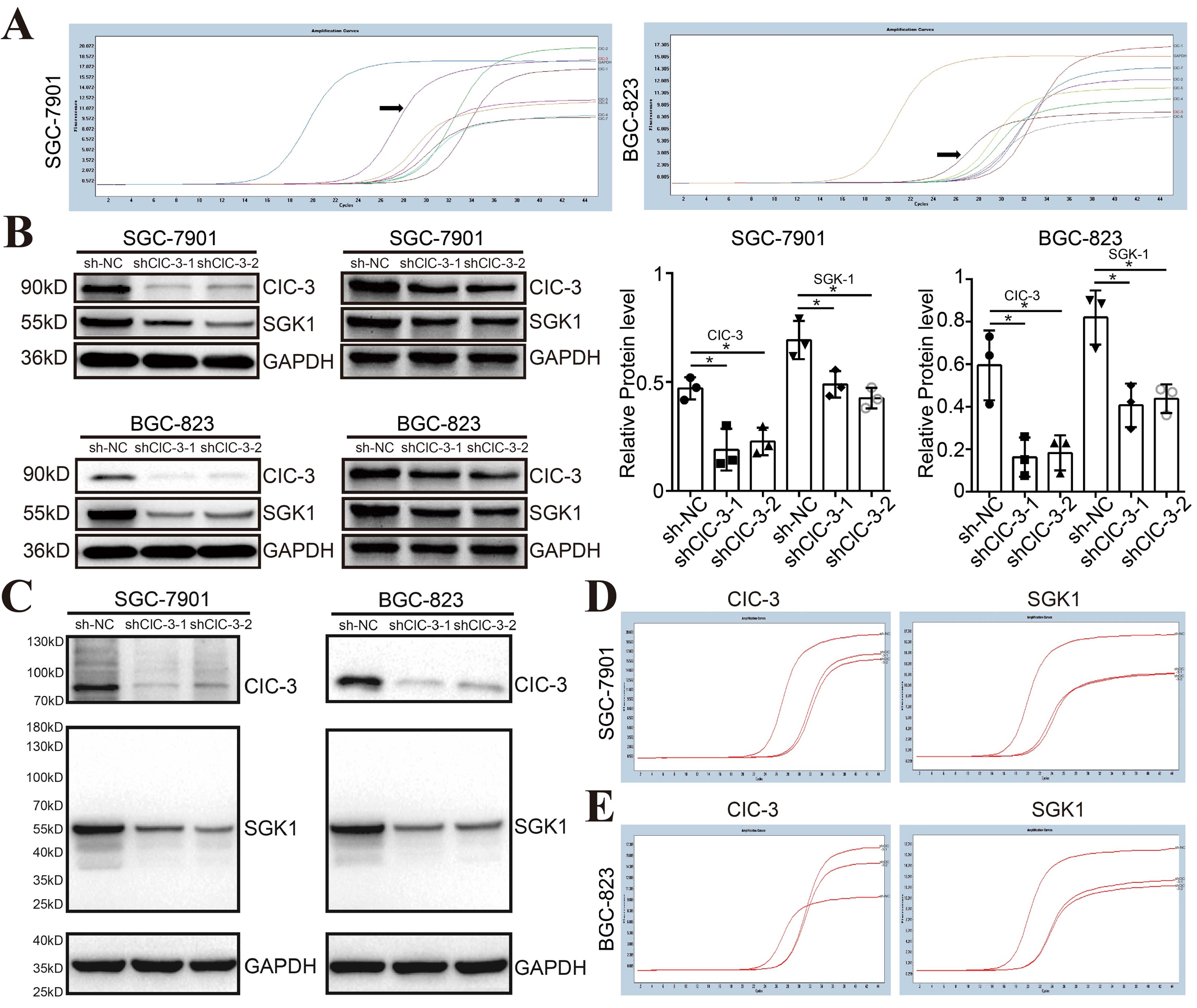

Supplement: Supplementary file 2 — Figure S2 [file 41419_2020_3107_MOESM2_ESM.jpg]

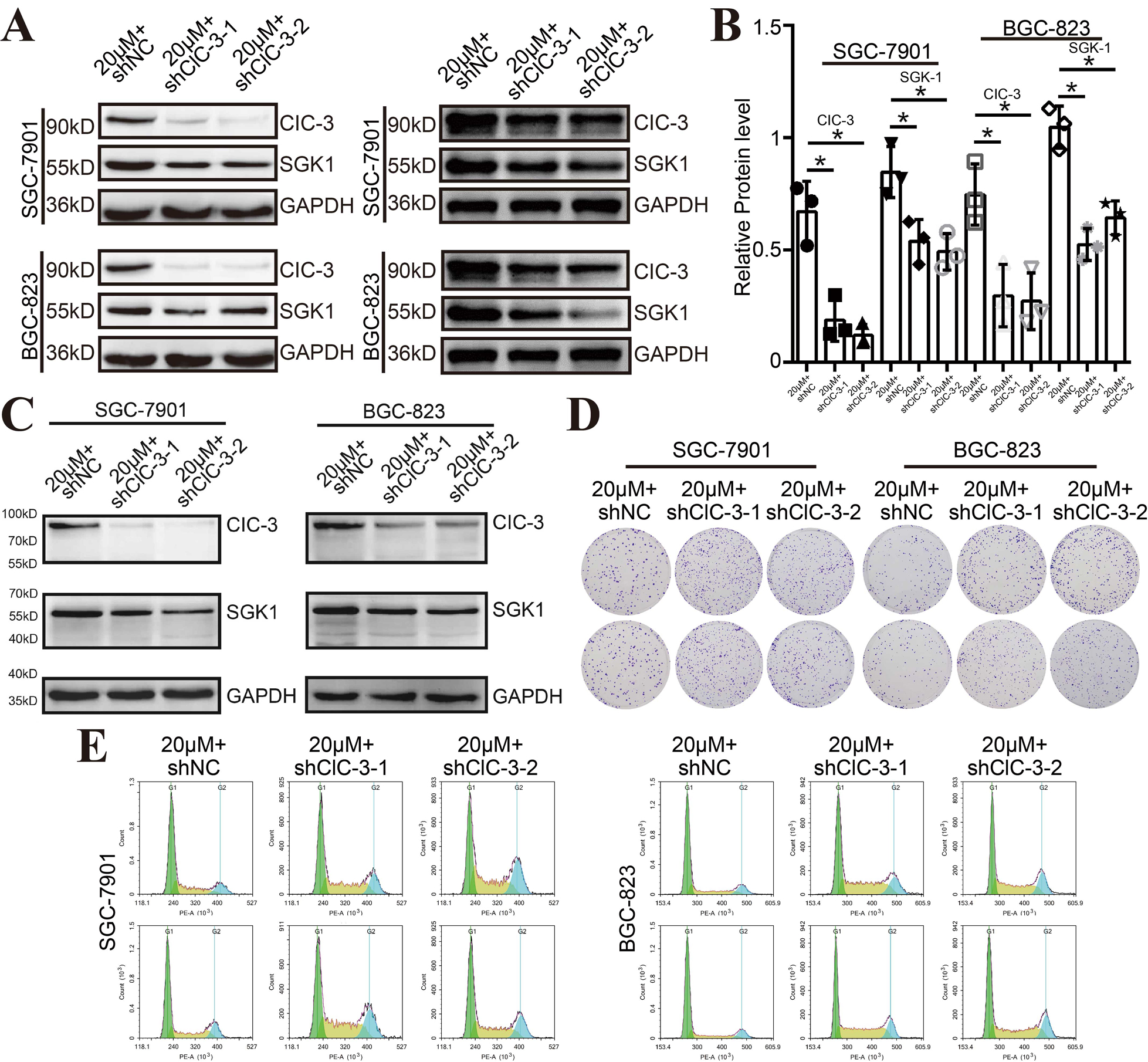

Supplement: Supplementary file 3 — Figure S3 [file 41419_2020_3107_MOESM3_ESM.jpg]

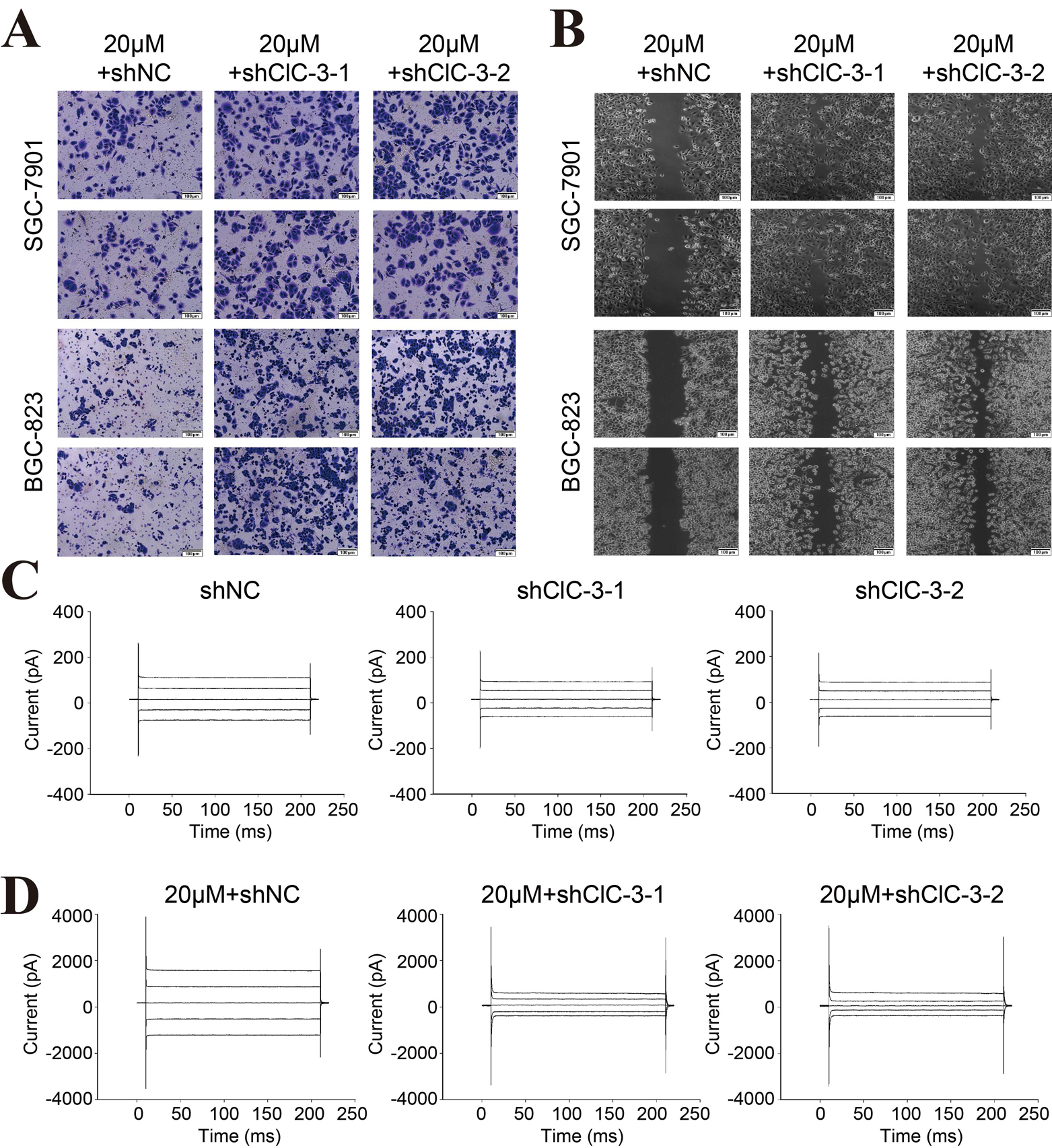

Supplement: Supplementary file 4 — Figure S4 [file 41419_2020_3107_MOESM4_ESM.jpg]

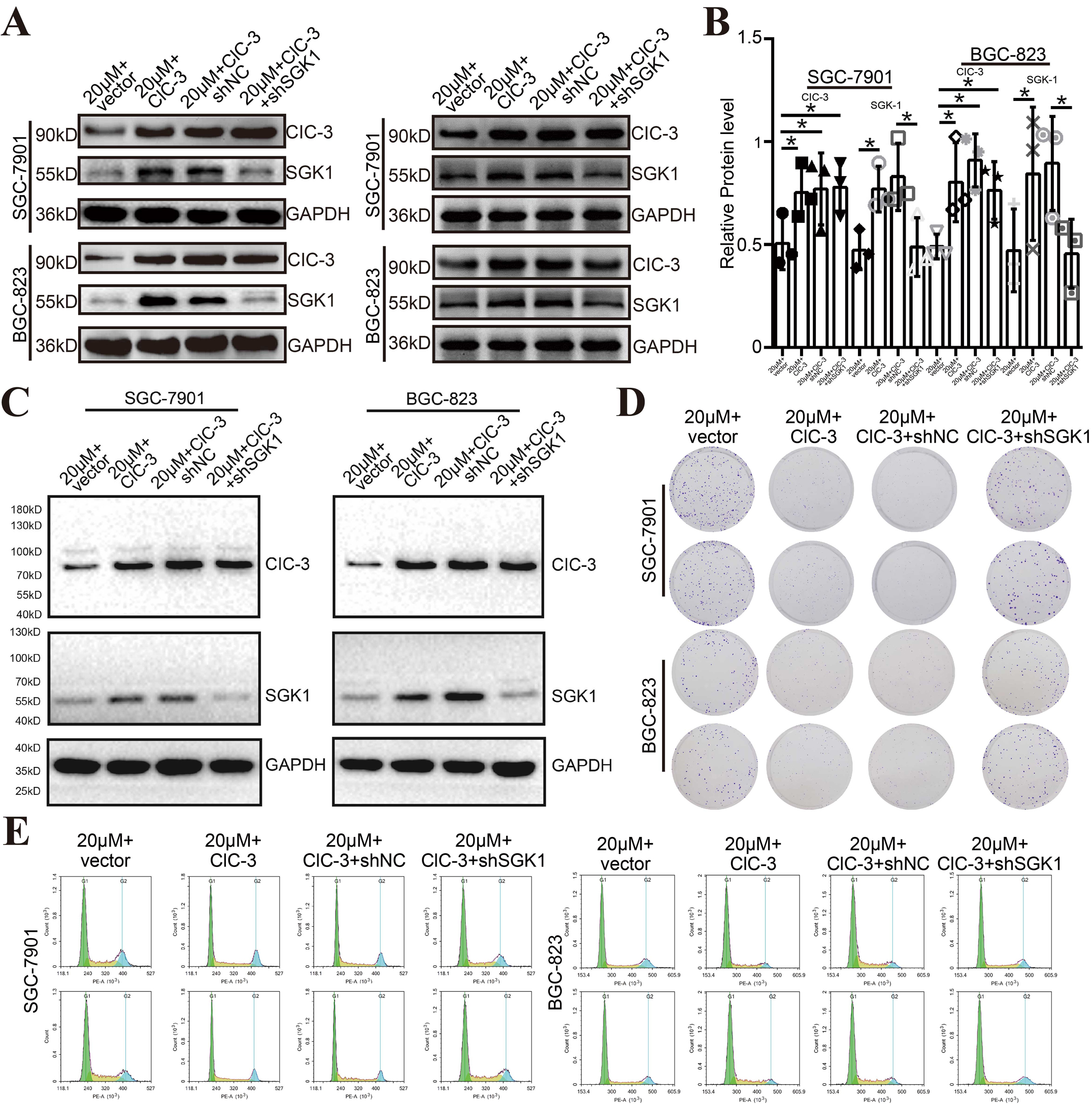

Supplement: Supplementary file 5 — Figure S5 [file 41419_2020_3107_MOESM5_ESM.jpg]

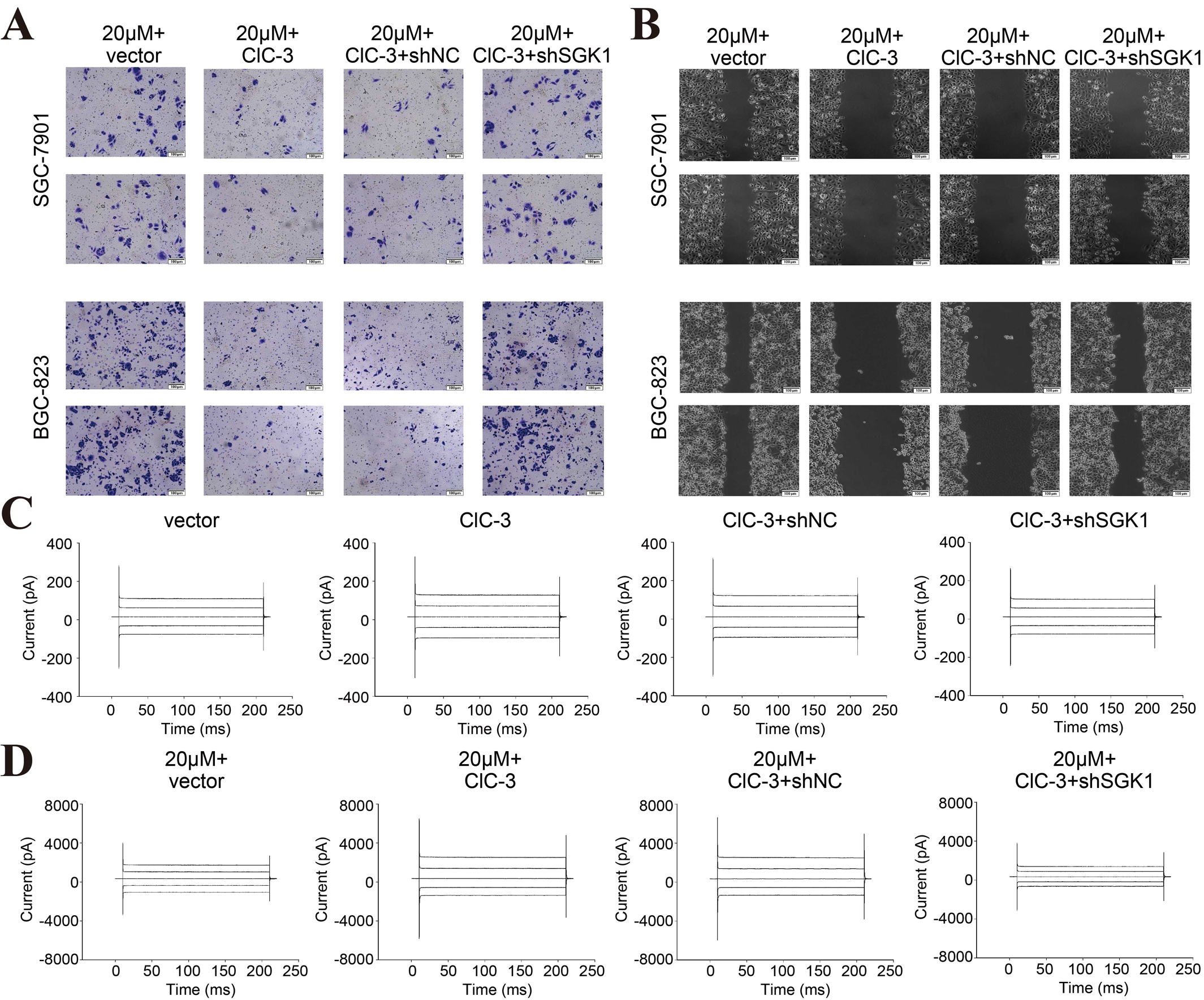

Supplement: Supplementary file 6 — Figure S6 [file 41419_2020_3107_MOESM6_ESM.jpg]

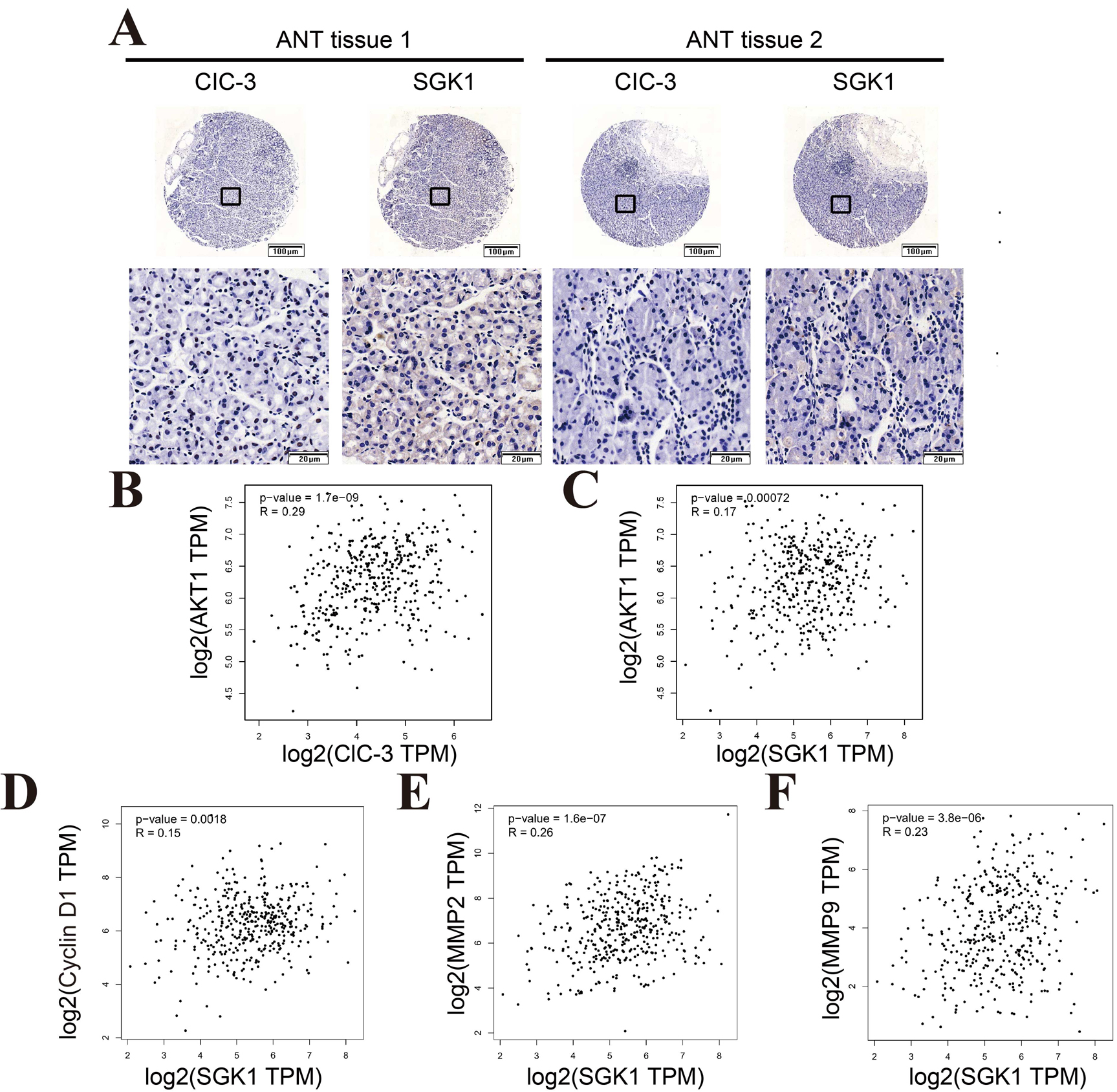

Supplement: Supplementary file 7 — Figure S7 [file 41419_2020_3107_MOESM7_ESM.jpg]

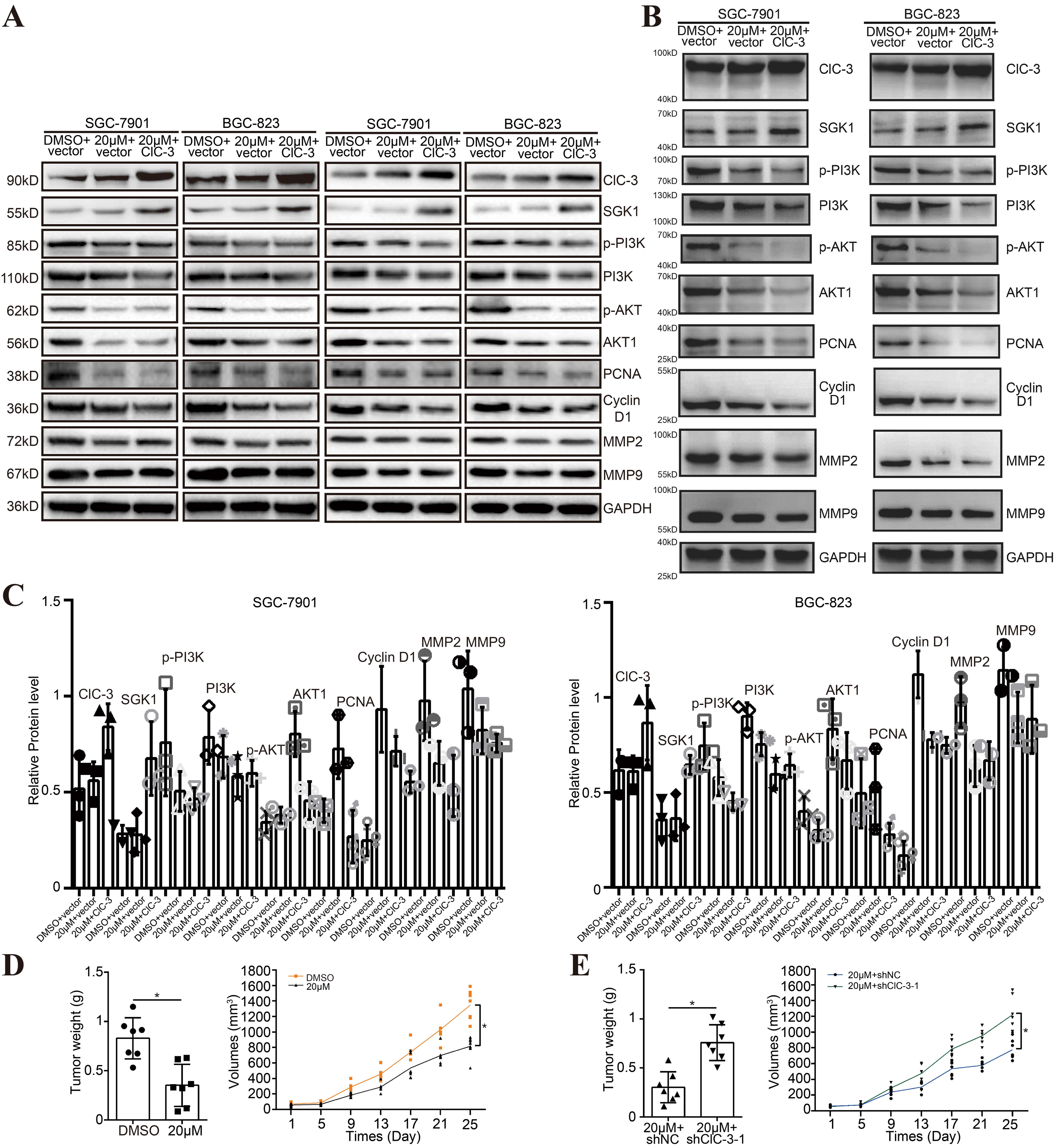

Supplement: Supplementary file 8 — Figure S8 [file 41419_2020_3107_MOESM8_ESM.jpg]
