# Supplementary material for: ClC-3/SGK1 regulatory axis enhances the olaparib-induced antitumor effect in human stomach adenocarcinoma
Source: Cell Death Dis. 2020 Oct 22;11(10):898. doi: 10.1038/s41419-020-03107-3 (PMC7583252; doi:10.1038/s41419-020-03107-3)
Supplement: Supplementary file 9 — Table S1 [file 41419_2020_3107_MOESM9_ESM.docx]

**Table S1** The qRT-PCR was designed with primer pairs as follows.

| **Gene** | **Forward (5’-3’)** | **Reverse (5’-3’)** |
| --- | --- | --- |
| **ClC-1** | GAATCCCCGAAATGAAGACA | TCCTACCAGCCTTCCAAATG |
| **ClC-2** | GCTGTCATTGGTATTGCTAGTGG | AGCGTCTCTTTCTGTGAGAGCTGT |
| **ClC-3** | TTGCCTACTATCACCACGAC | GCATCTCCAACCCATTTACT |
| **ClC-4** | CCCTGGTACATGGCTGAACT | CTCTGGCGTGTGTAGGGATT |
| **ClC-5** | TGGACTCCTCCAAGCTCTGT | AGGCCAGAAGGGATCTTCAT |
| **ClC-6** | ATTTGGGTTTCTTCGTCGTG | CGGCATTCTCCTAACACCAT |
| **ClC-7** | GGAGAAAATGGCCTACACGA | AGATCAGCACGAAGGCAACT |
| **GAPDH** | GGTGGTCTCCTCTGACTTCAACA | GTTGCTGTAGCCAAATTCGTTGT |
| **SGK1** | AGGATGGGTCTGAACGACTTT | GCCCTTTCCGATCACTTTCAAG |
