# Supplementary material for: ClC-3/SGK1 regulatory axis enhances the olaparib-induced antitumor effect in human stomach adenocarcinoma
Source: Cell Death Dis. 2020 Oct 22;11(10):898. doi: 10.1038/s41419-020-03107-3 (PMC7583252; doi:10.1038/s41419-020-03107-3)
Supplement: Supplementary file 11 — Supplementary Figure Legends [file 41419_2020_3107_MOESM11_ESM.docx]

**Supplementary Figure Legends**

**Figure S1. Olaparib exerted antitumor effect in STAD cell lines.** (**a**) Olaparib inhibited the clonogenicity of STAD cells in a dose-dependent manner. (**b**) Olaparib arrested the cell cycle of STAD cells at the G0/G1 phase in a dose-dependent manner. (**c**) Olaparib inhibited the invasion of STAD cells in a dose-dependent manner. (**d**) Olaparib inhibited the migration of STAD cells in a dose-dependent manner.

**Figure S2. ClC-3/SGK1 regulatory axis was identified and validated in STAD cell lines.** (**a**) The representative amplification curve of qRT-PCR revealed that the RNA level of ClC-3 was significantly abundant compared with other ClC chloride channel superfamily members (indicated with the black arrow). (**b**) The protein levels of ClC-3 and SGK1 were significantly decreased in the two ClC-3 KD cells, and the quantified results were shown in the right panel. (**c**) The raw western blot images of the blots in figure 2f. shNC, negative control shRNA; shClC-3-1, ClC-3 knockdown shRNA-1; shClC-3-2, ClC-3 knockdown shRNA-2. (**d, e**) The representative amplification curve of qRT-PCR revealed that the RNA levels of ClC-3 and SGK1 were significantly reduced in the two ClC-3 KD cells. * *P*<0.05

**Figure S3. Down-regulation of ClC-3/SGK1 axis attenuated olaparib-induced cell growth inhibition.** (**a**) The protein levels of ClC-3 and SGK1 were significantly decreased in the two ClC-3 KD cells treated with 20 μM olaparib. (**b**) The quantified results of the protein levels in figure S3a were exhibited. (**c**) The raw western blot images of the blots in figure 3a. shNC, negative control shRNA; shClC-3-1, ClC-3 knockdown shRNA-1; shClC-3-2, ClC-3 knockdown shRNA-2. (**d**) Down-regulation of ClC-3/SGK1 axis attenuated olaparib-induced cell clonogenicity inhibition. (**e**) Down-regulation of ClC-3/SGK1 axis attenuated olaparib-induced cell cycle arrest at the G0/G1 phase. * *P*<0.05

**Figure S4. Down-regulation of ClC-3/SGK1 axis attenuated olaparib-induced cell migration inhibition.** (**a**) Down-regulation of ClC-3/SGK1 axis attenuated olaparib-induced cell invasion inhibition. (**b**) Down-regulation of ClC-3/SGK1 axis attenuated olaparib-induced cell migration inhibition. (**c**) Typical current traces of the chloride current recorded by whole-cell patch clamp. The instantaneous basal chloride current results indicated that there was no significant difference between ClC-3 NC and ClC-3 KD SGC-7901 cells. (**d**) Typical current traces of the chloride current recorded by whole-cell patch clamp. The instantaneous chloride current activated by olaparib was decreased in ClC-3 KD SGC-7901 cells. * *P*<0.05

**Figure S5. Up-regulation of ClC-3/SGK1 axis enhanced olaparib-induced cell growth inhibition.** (**a**) The protein levels of ClC-3 and SGK1 were significantly increased in the two ClC-3 OV cells treated with 20 μM olaparib. (**b**) The quantified results of the protein levels in figure S5a were exhibited. (**c**) The raw western blot images of the blots in figure 5a. vector, empty vector-transfected cells; ClC-3, ClC-3 overexpression cells; ClC-3+shNC, ClC-3 overexpression and SGK1 negative control cells; ClC-3+shSGK1, ClC-3 overexpression and SGK1 knockdown cells. (**d**) Up-regulation of ClC-3/SGK1 axis enhanced olaparib-induced cell clonogenicity inhibition, and the enhancement effect could be attenuated by SGK1 KD. (**e**) Up-regulation of ClC-3/SGK1 axis enhanced olaparib-induced cell cycle arrest at the G0/G1 phase, and the enhancement effect could be attenuated by SGK1 KD. * *P*<0.05

**Figure S6. Up-regulation of ClC-3/SGK1 axis enhanced olaparib-induced cell migration inhibition.** (**a**) Up-regulation of ClC-3/SGK1 axis enhanced olaparib-induced cell invasion inhibition, and the enhancement effect could be attenuated by SGK1 KD. (**b**) Up-regulation of ClC-3/SGK1 axis enhanced olaparib-induced cell migration inhibition, and the enhancement effect could be attenuated by SGK1 KD. (**c**) Typical current traces of the chloride current recorded by whole-cell patch clamp. The instantaneous basal chloride current results indicated that there was no significant difference among ClC-3 vector, ClC-3 OV, ClC-3 OV+SGK1 NC, and ClC-3 OV+SGK1 KD SGC-7901 cells. (**d**) Typical current traces of the chloride current recorded by whole-cell patch clamp. The instantaneous chloride current activated by olaparib was increased in ClC-3 OV SGC-7901 cells, and the increase effect could be attenuated by SGK1 KD. * *P*<0.05

**Figure S7. ClC-3 and SGK1 were lowly expressed in ANTs, and the TPM of ClC-3/SGK1 and AKT1 was positively correlated in STAD tissues.** (**a**) Representative images of ClC-3 and SGK1 protein expression in adjacent normal tissues (ANTs). (**b**) The transcript per million (TPM) expression of ClC-3 and AKT1 was positively correlated in STAD tissues. (**c**) The TPM expression of SGK1 and AKT1 was positively correlated in STAD tissues. (**d, e, f**) The TPM expression of SGK1 and Cyclin D1/MMP2/MMP9 was positively correlated in STAD tissues.

**Figure S8. Olaparib inhibited the downstream PI3K/AKT** **pathway of ClC-3/SGK1 axis and exerted antitumor effect *in vivo*.** (**a**) Olaparib inhibited the PI3K/AKT pathway in STAD cells, and up-regulation of ClC-3/SGK1 axis enhanced olaparib-induced PI3K/AKT pathway inhibition. (**b**) The raw western blot images of the blots in figure 8a. vector, empty vector-transfected cells; ClC-3, ClC-3 overexpression cells. (**c**) The quantified results of the protein levels in figure S8a were exhibited. (**d, e**) Down-regulation of ClC-3 attenuated olaparib-induced tumor weight reduction and volume decrease. * *P*<0.05
