# Supplementary material for: ClC-3/SGK1 regulatory axis enhances the olaparib-induced antitumor effect in human stomach adenocarcinoma
Source: Cell Death Dis. 2020 Oct 22;11(10):898. doi: 10.1038/s41419-020-03107-3 (PMC7583252; doi:10.1038/s41419-020-03107-3)
Supplement: Supplementary file 12 — Supplementary Table Legends [file 41419_2020_3107_MOESM12_ESM.docx]

**Supplementary Table Legends**

**Table S1. The qRT-PCR was designed with primer pairs as follows.** In Table S1, the primers for qRT-PCR were shown, respectively.

**Table S2. 60 same genes were down-regulated in the two ClC-3 KD cells.** In Table S2, the 60 down-regulated same genes were exhibited in the two ClC-3 KD cells, of which the SGK1 gene is the mostly down-regulated.
